# Gastrointestinal perforation: clinical and MDCT clues for identification of aetiology

**DOI:** 10.1186/s13244-019-0823-6

**Published:** 2020-02-21

**Authors:** Styliani Pouli, Androniki Kozana, Ioanna Papakitsou, Maria Daskalogiannaki, Maria Raissaki

**Affiliations:** 1 Department of Radiology, University Hospital of Heraklion, Faculty of Medicine-University of Crete, Stavrakia, Voutes 21110, Heraklion, Crete, Greece; 2 Department of Internal Medicine, University Hospital of Heraklion, Heraklion, Greece

**Keywords:** Gastrointestinal perforation, Emergency, Aetiology, Multidetector computed tomography

## Abstract

Gastrointestinal tract (GIT) perforation is a common medical emergency associated with considerable mortality, ranging from 30 to 50%. Clinical presentation varies: oesophageal perforations can present with acute chest pain, odynophagia and vomiting, gastroduodenal perforations with acute severe abdominal pain, while colonic perforations tend to follow a slower progression course with secondary bacterial peritonitis or localised abscesses. A subset of patients may present with delayed symptoms, abscess mimicking an abdominal mass, or with sepsis.

Direct multidetector computed tomography (MDCT) findings support the diagnosis and localise the perforation site while ancillary findings may suggest underlying conditions that need further investigation following primary repair of ruptured bowel. MDCT findings include extraluminal gas, visible bowel wall discontinuity, extraluminal contrast, bowel wall thickening, abnormal mural enhancement, localised fat stranding and/or free fluid, as well as localised phlegmon or abscess in contained perforations.

The purpose of this article is to review the spectrum of MDCT findings encountered in GIT perforation and emphasise the MDCT and clinical clues suggestive of the underlying aetiology and localisation of perforation site.

## Key points

- GIT perforation manifests with extraluminal gas, wall discontinuity or thickening, fat stranding.
- Pneumoperitoneum and extraluminal oral contrast range from abundant to absent.
- Ancillary findings comprise foreign bodies, masses, excessive wall thickening, ischemia, faecal impaction.
- Supramesocolic pneumoperitoneum and hyperenhancing gastroduodenal wall suggest perforated peptic ulcer disease.
- Persistent or increasing free air and/or ascites postoperatively indicate iatrogenic perforation.

## Background

Despite progress in emergency medicine, gastrointestinal tract perforation remains a condition associated with considerable mortality, ranging from 30 to 50% [1, 2]. Clinical presentation varies: oesophageal perforations can present with non-specific symptoms such as acute chest pain, odynophagia and vomiting [2], gastroduodenal perforations typically present with acute abdominal pain [1, 3], whereas colonic perforations tend to follow a slower progression course, presenting with secondary bacterial peritonitis or localised abscess formation [1, 4]. A subset of patients exhibits delayed symptoms, abscess formation that mimics an abdominal mass, or with sepsis [3]. Evaluation of patients with abdominal, chest or neck pain comprises a thorough medical history, inquiring about prior bouts of similar pain and predisposing conditions such as prior surgery or instrumentation, abdominal trauma, ingested foreign bodies, medical conditions including peptic ulcer disease and medications, especially non-steroid anti-inflammatory drugs (NSAIDs). Management may entail a short-term treatment of the cause, i.e. retrieval of an ingested foreign body or a long-term treatment of a medical condition such as Crohn’s disease (CD) complicated by perforation.

Multidetector computed tomography (MDCT) is the modality of choice for the evaluation of suspected perforation, due to its high sensitivity in detecting extraluminal gas and ability to localise the perforation site, with an accuracy ranging from 82 to 90% [2, 5–8].

In this review, we discuss the spectrum of MDCT findings encountered in GIT perforation and emphasise the imaging and clinical clues that may be important for prompt diagnosis of the aetiology of perforation and for localisation of the perforation site.

## CT technique

In suspected pharyngeal and oesophageal perforation, the thorax should be scanned from the oropharynx and thoracic inlet, respectively, to the upper abdomen [2]. For suspected gastroduodenal, small or large bowel perforation, scanning should extend from the lung bases to the pubic symphysis [1, 7–10].

Axial images of 2-mm slice thickness should be obtained at a non-contrast and a portal phase (100 mL, 70–80 s following intravenous administration of low-osmolarity iodinated contrast medium) [1, 6–12]. In suspected ischemic infarction, a biphasic technique in the arterial and portal phases, following 120–150 mL of contrast, is required for the detection of vascular changes and perfusion abnormalities [3]. In blunt trauma, an added late 3–5-min phase is needed to exclude low-flow active bleeding [3, 13]. Review of images in multiple window settings is indispensable, since extraluminal air and foreign bodies are better visualised in lung and bone window settings, respectively [2, 6–12, 14–18]. Multiplanar reformations can greatly increase the accuracy in localisation of perforation site [2, 6–12, 14–18].

The administration of water-soluble iodinated oral contrast remains debatable. Oral contrast can significantly delay management, obscure radiopaque foreign bodies and may be poorly tolerated [5, 7–9, 11, 16, 19, 20]. When present, oral contrast leakage is a highly specific sign for perforation site localisation; however, it carries a low sensitivity (19–42%) [2, 10, 12–14, 20].

Evaluation of colorectal perforation following contrast per os requires a preparation time of 3 h, especially in patients with diminished bowel motility [11]; thus, some institutions recommend administration of intrarectal contrast [10, 11, 13]. This should be performed cautiously and in small quantities to avoid further rupture of a friable colonic wall (i.e. in inflammatory, ischemic or neoplastic conditions). In the presence of a known low colorectal or coloanal anastomosis, the use of a rectal tube is advised instead of a balloon catheter, to avoid disruption of the anastomosis [10].

In our institution oral contrast is generally avoided when perforation is strongly suspected. Oral contrast is considered in patients with good clinical condition which allows the delay in performing the scan related to bowel preparation, especially in atypical clinical findings with a wide differential diagnosis, in a complicated history such as suspected abscess, operated or oncologic patients and when the initial non-contrast scan provides inconclusive findings that could be better characterised using oral contrast. The above are particularly applicable for oesophageal and gastroduodenal perforations. Intrarectal contrast is reserved for challenging occasions, such as in post-operative and post-radiotherapy patients.

## CT findings

Direct CT signs independent of perforation site include extraluminal gas and extraluminal oral contrast, as well as bowel wall discontinuity from which contrast, air or luminal contents are spilled out [2, 5–8, 10, 12, 15]. Bowel discontinuity does not invariably cause pneumoperitoneum and may result into a localised phlegmon or an abscess [2, 7, 8, 10, 12]. Indirect signs include segmental bowel wall thickening, abnormal wall enhancement, localised fat stranding and/or free fluid [2, 5–8, 10, 12, 15]. It is important, when possible, to distinguish “contained” from “free” perforations because in the latter case emergent surgery is required [3]. Aetiology-specific signs are analysed in upcoming sections.

## Oesophageal perforations

Oesophageal perforation represents a rare but life-threatening condition [21–23]. Overall mortality approaches 13.3% but rises significantly after 24 h from symptom onset [21, 23, 24].

Most patients present with significant distress accompanied by non-specific symptomatology which comprises sudden-onset pain, fever, dysphagia, dyspnea, hoarseness, dysphonia and subcutaneous emphysema in various combinations [2, 21, 23].

CT findings include oesophageal wall thickening, mural gas, mural defect(s), mediastinal or cervical fat stranding, free gas/contrast/fluid in the mediastinum, subcutaneous emphysema, pleural effusion and foreign bodies [2, 12]. In cases of complicated tumours or strictures, an oesophago-respiratory fistula, lung abscess or recurrent aspiration pneumonia may develop [2]. Lower oesophageal perforations may result in extraluminal gas that dissects inferiorly into the abdomen and can mimic gastric perforation [7, 10].

The most frequent causes of oesophageal perforation are iatrogenic, followed by spontaneous rupture, foreign body ingestion, trauma, tumour and less commonly caustic ingestion, pill oesophagitis, and infectious ulcers in patients with AIDS [2, 21, 23–26].

**Fig. 1:**
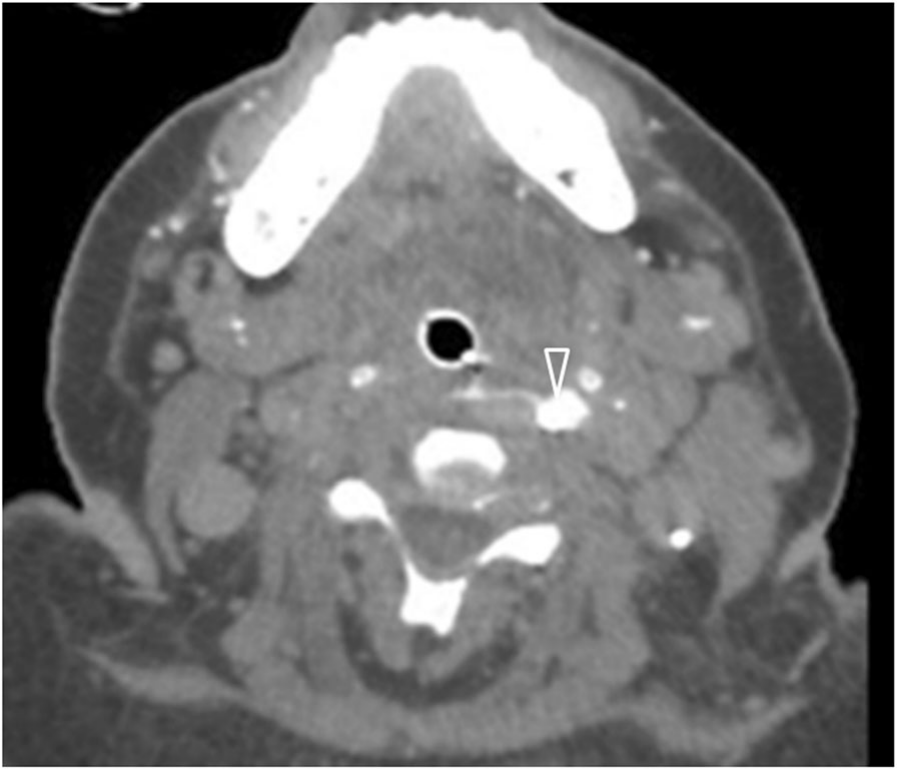
75-year-old patient with pharyngeal perforation following repeated nasogastric tube insertions. Axial contrast-enhanced image of the neck following oral contrast administration demonstrates a contrast leak through the left aspect of the pharyngeal wall (arrowhead)

Iatrogenic manipulations constitute the leading cause of oesophageal perforation [1, 21, 24] (Fig. 1). The risk of oesophageal injury is highest with rigid endoscopies (0.11%), escalating up to 10–15% with the addition of treatment manipulations, such as stricture dilatation and stent placement [2, 21]. Endoscopic retrograde cholangiopancreatography (ERCP) carries low perforation rates (0.03–0.3%); the oesophagus is the most common associated perforation site, affected in approximately half of perforations [2].

**Fig. 2:**
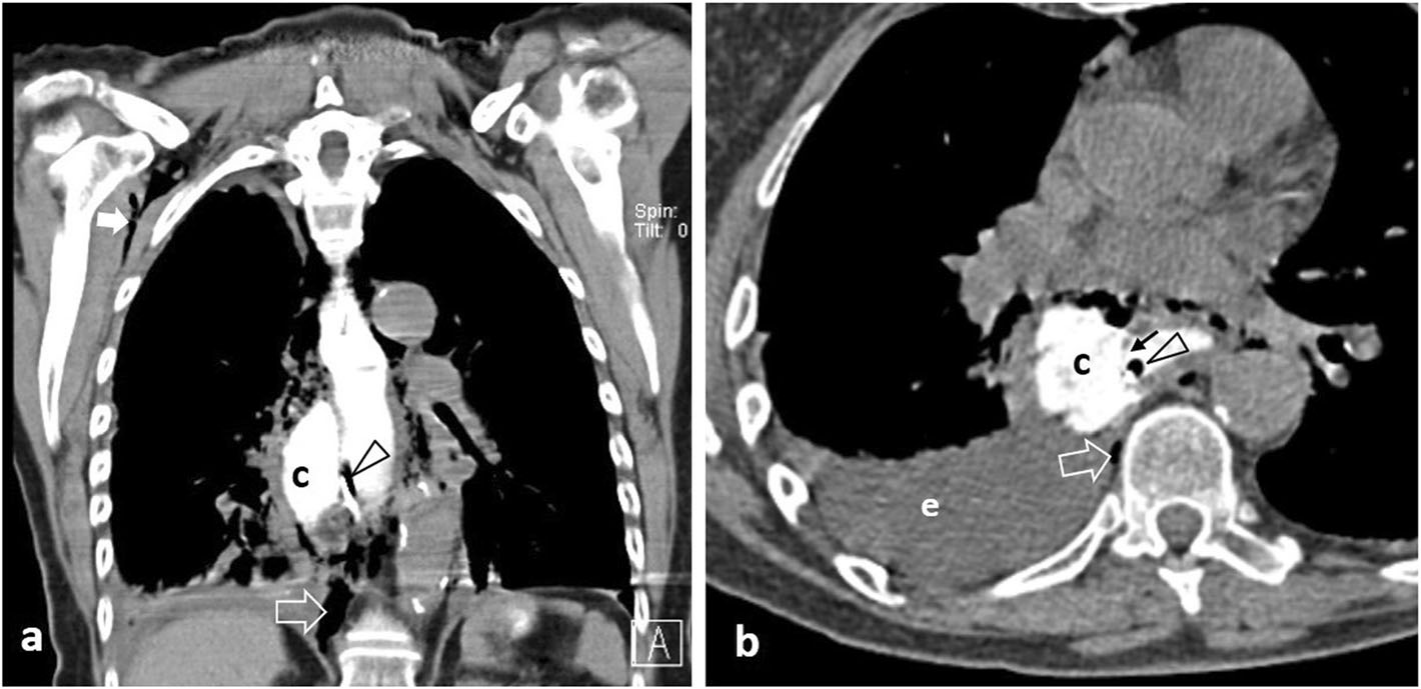
67-year-old patient with Boerhaave syndrome. **a** Coronal and (**b**) axial unenhanced images following oral contrast administration. A large mural defect (black arrow) results in contrast leaking into a paraoesophageal collection (c). Note multiple gas bubbles anteroposterior to the oesophagus, retrocrurally (open arrow), at the right axilla (white arrow) and a right-sided pleural effusion (e). A nasogastric tube (arrowhead) is noted

**Fig. 3:**
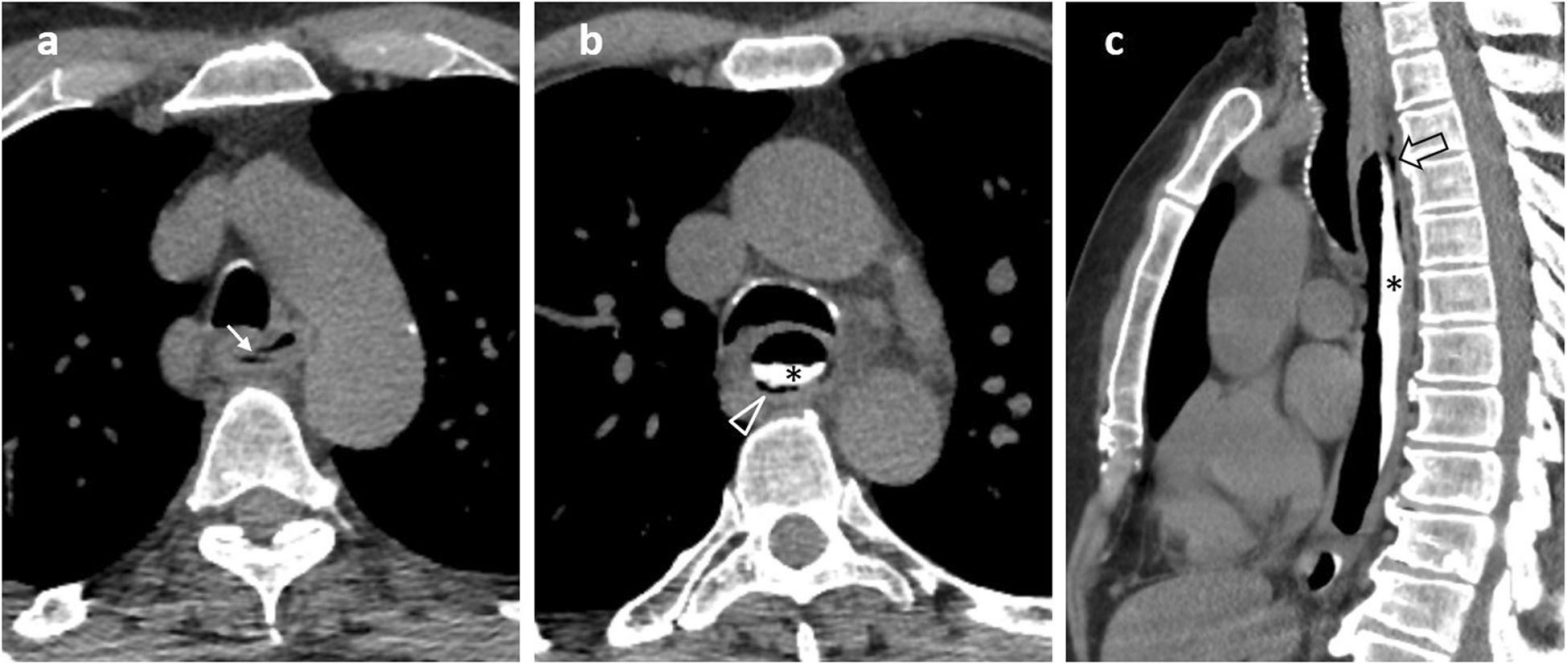
75-year-old patient with oesophageal intramural rupture. **a** Axial unenhanced image demonstrates blurring of paraoesophageal fat, a double air-filled lumen and an interposed diaphragm (arrow) consistent with a flap. **b** Axial and (**c**) sagittal unenhanced images following oral contrast administration demonstrate contrast at the dependent part of the lumen (*) and a submucosal curvilinear collection of gas (arrowhead) better demonstrated on the sagittal plane (open arrow), giving the oesophagus a double-barreled appearance

Spontaneous oesophageal perforation (Boerhaave syndrome) is caused by incomplete cricopharyngeal relaxation during severe vomiting, resulting in abruptly increased intraluminal pressure with subsequent rupture [2, 27, 28]. Rupture occurs usually at the distal left posterior oesophageal wall, accompanied by pneumomediastinum and left pleural effusion (Fig. 2). Vomiting, chest pain and subcutaneous emphysema comprise the Mackler triad which is indicative of this syndrome [2, 23]. An intermediate form of oesophageal injury referred to as submucosal dissection or intramural rupture causing intramural air or contrast leak is rare (Fig. 3), associated with instrumentation, foreign body impaction and forceful vomiting [28].

**Fig. 4:**
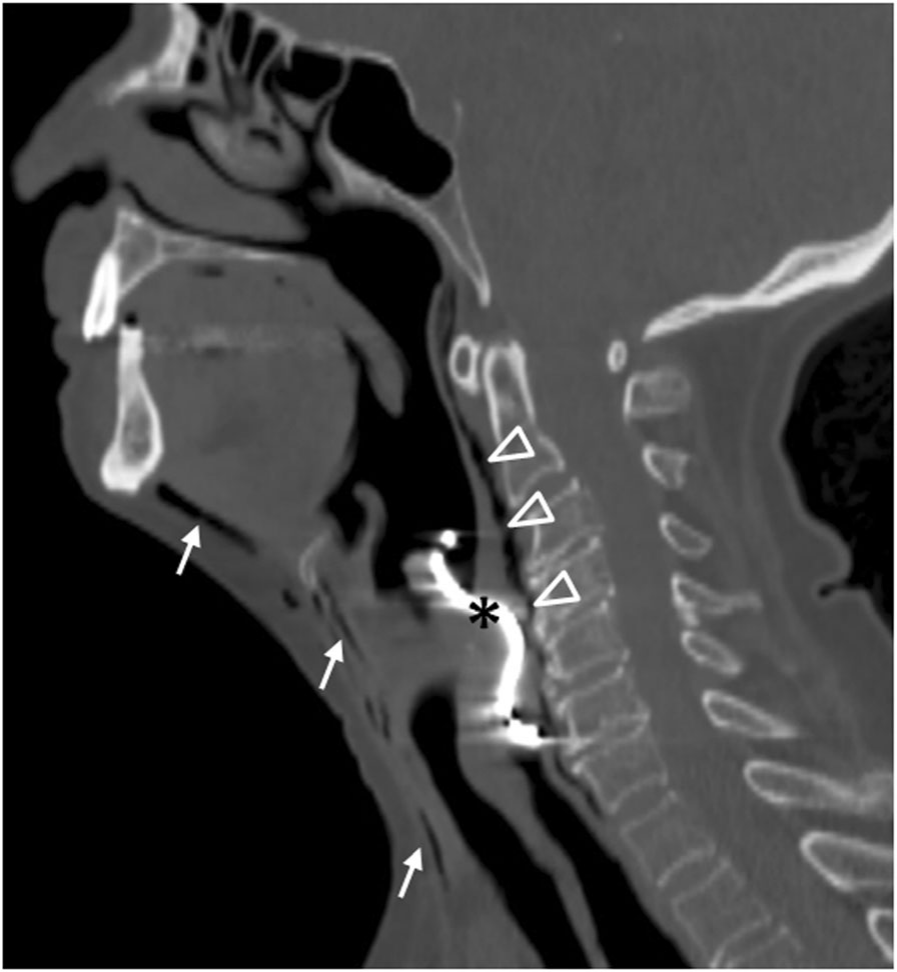
77-year-old patient with prolonged dysphagia. Sagittal reformatted image in bone window demonstrates a swallowed perforating denture (*) impinging on the upper oesophagus. There is superficial (arrows) and deep (arrowheads) cervical emphysema

Food boluses are the most common foreign bodies to cause perforation, through impaction that causes wall ischemia and subsequent necrosis [2]. Fish and chicken bones usually directly perforate the oesophageal wall. Foreign bodies should be considered when children, elderly and mentally handicapped patients present with prolonged dysphagia (Fig. 4).

The thin oesophageal wall and its poor arterial supply make it particularly susceptible to traumatic rupture, with mortality rates greater than 20% [21]. A high index of suspicion is warranted whenever there are penetrating injuries in the cervical and thoracic region, since oesophageal damage can be easily missed during clinical examination [2, 23].

Perforation in the setting of oesophageal carcinoma is rare, usually following radiotherapy, iatrogenic instrumentation, or from pressure necrosis due to a previously placed stent [2].

## Gastroduodenal perforations

Gastric and duodenal perforations share similar causative mechanisms and clinical presentation. Patients with gastric or intraperitoneal duodenal perforation usually present with acute abdominal pain, guarding and rebound tenderness due to rapidly evolving chemical peritonitis caused by the effect of acidic/biliary/pancreatic contents on the peritoneal cavity [1, 3, 15]. Immunosuppressed patients, including those treated with steroid drugs may present with non-specific symptomatology following contained or retroperitoneal perforation [3]. MDCT can demonstrate gastric and duodenal perforation with an accuracy greater than 90% [9]. Gas in the supramesocolic space indicates gastroduodenal perforation. Due to anatomic proximity, free gas bubbles in the lesser sac are indicative of perforation at the posterior gastric wall and at the duodenal bulb [2, 3, 8, 10, 27]. Gas bubbles along the falciform ligament (falciform ligament sign) and in the intrahepatic fissure of ligamentum teres (ligamentum teres sign) are useful predictors of intraperitoneal gastroduodenal perforation [3, 5, 8, 10, 12, 15, 27]. Pneumoperitoneum may range from abundant to absent [2, 3, 7, 8]. Perforation of the retroperitoneal duodenum characteristically causes pneumoretroperitoneum in the right anterior pararenal space [2, 10]. CT images should also be scrutinised for signs of oral contrast extravasation, focal thinning/discontinuity of the gastroduodenal wall, segmental wall thickening, perigastric or periduodenal fluid and adjacent fat stranding [3, 10, 27, 29]. Gas extending to the mediastinum may be encountered.

Peptic ulcer disease (PUD) remains the leading cause of gastroduodenal perforation, followed by trauma, malignancy and iatrogenic injuries [2, 7, 8, 15, 30, 31]. Less common causes include inflammatory and ischemic conditions like mesenteric infarction, volvulus, intussusception and vasculitis [15, 32].

**Fig. 5:**
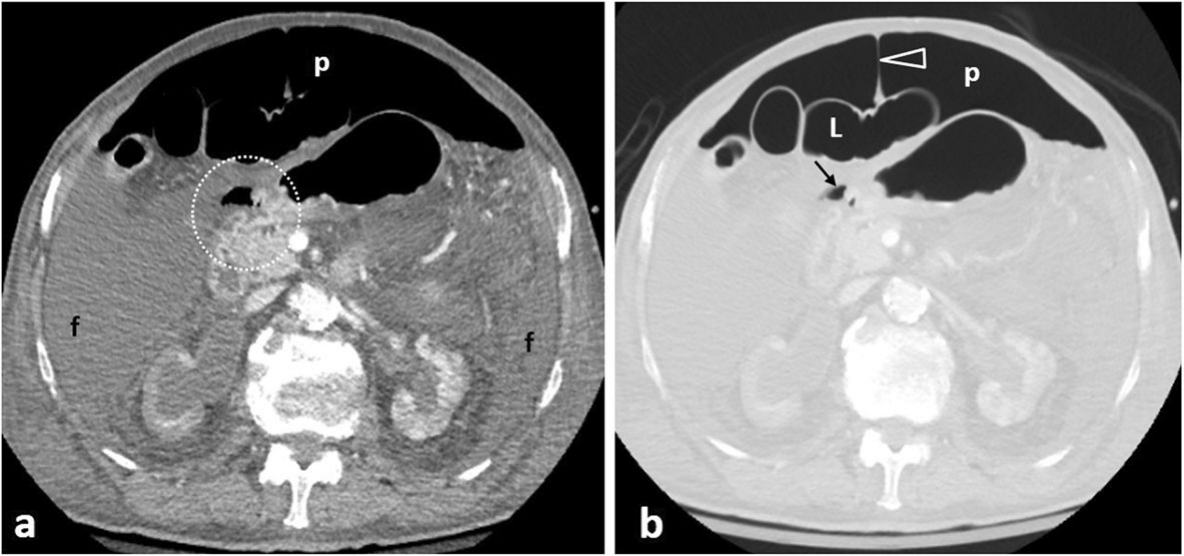
92-year-old patient on long-term corticosteroids. **a** Soft tissue window and (**b**) lung window contrast-enhanced axial images demonstrate a discontinuity of the hyperenhancing gastric mucosa, postero-medially to an extraluminal gas bubble (area within circle). Free fluid (f) and pneumoperitoneum (p) are outlining air-filled bowel loops (L) and the falciform ligament (arrowhead). The triangular-shaped separate gas bubble (arrow) points towards the perforation site

**Fig. 6:**
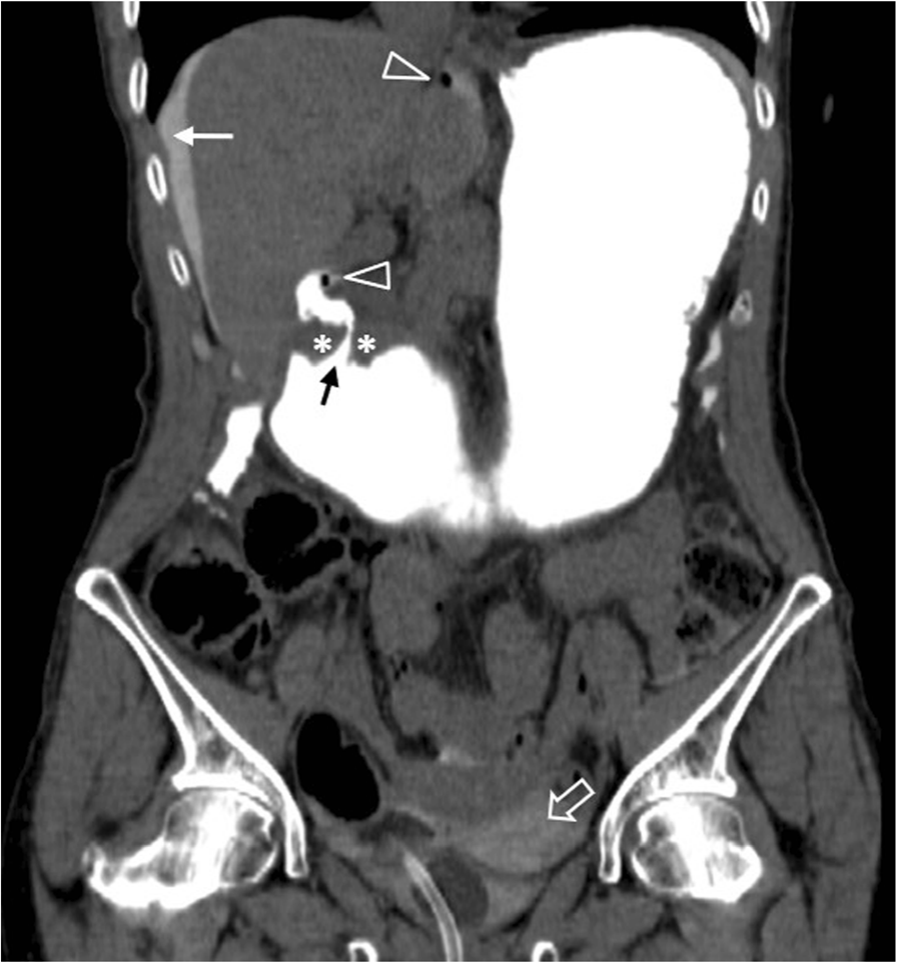
65-year-old patient with perforated duodenal ulcer. Coronal unenhanced image (modified soft tissue window) following oral contrast administration demonstrates a thickened duodenal wall (*), contrast leaking (black arrow) to the peritoneal spaces and free gas bubbles (arrowheads). Note hyperdensity of perihepatic free fluid (white arrow) compared with the diluted contrast in the rectouterine pouch (open arrow)

Perforation is encountered in 5–20% of PUD cases [2], contributing to 70% of ulcer-related mortality [32]. Associated risk factors include *Helicobacter pylorii* infection NSAIDs, acetylsalicylic acid, corticosteroids (Fig. 5), bevacizumab, stress, tobacco, alcohol abuse, and less commonly inflammatory bowel disease and Zollinger-Ellison syndrome [31, 33–35]. Ulcers along the anterior wall and curvatures perforate directly into the peritoneal space (Fig. 6), while ulcers along the posterior wall or duodenum tend to result in contained perforations [3, 27, 29, 33]. The gastric antrum and duodenal bulb are the most common perforation sites in ulcerative disease [2, 3, 31]. Post-bulbar ulcers are rare (3–5%) and should raise suspicion for conditions like Zollinger-Ellison syndrome or Crohn’s [35].

The retroperitoneal duodenum is the most common site affected in blunt trauma due to its firm attachment and proximity to the vertebral column [2, 3, 8]. CT is helpful in distinguishing a duodenal hematoma, which is associated with wall thickening and surrounding fluid, from a duodenal perforation which may manifest with gas and/or extravasated oral contrast in the right anterior pararenal space in addition to the above findings [10, 36]. Gastric injury should be suspected in the presence of splenic, diaphragmatic or left hemiliver injuries [2, 29]. Since the stomach usually collapses following rupture, identifying the injury tract on CT may be the only clue, suggesting penetrating injury [2, 29]. In the setting of concomitant left diaphragmatic injury, leakage of gastric contents into the overlying chest cavity may result in empyema [2].

**Fig. 7:**
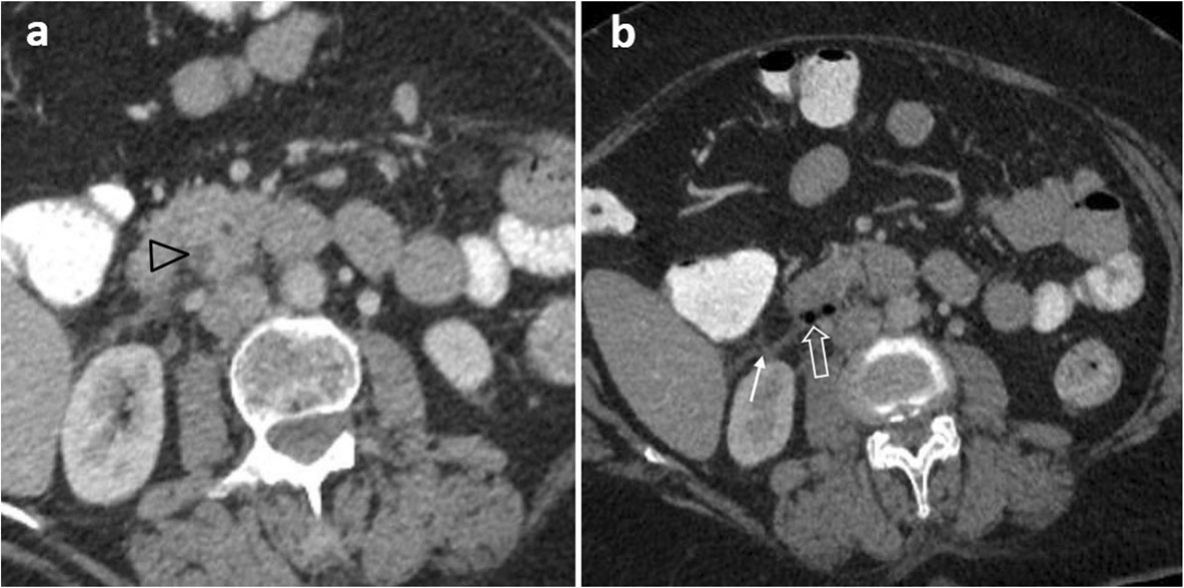
75-year-old patient with duodenal perforation post-ERCP. **a, b** Axial contrast-enhanced image shows a focal discontinuity of wall enhancement (arrowhead), associated with retroperitoneal gas bubbles (open arrow) posterior to the 3rd duodenal segment and thickening of Gerota’s fascia (white arrow)

Iatrogenic perforations occur rarely following ERCP (0.03–0.3%) (Fig. 7), oesophagogastroduodenoscopy, inferior vena cava filter and biliary stent placement [2, 3]. ERCP-related duodenal perforations have been classified in four categories, depending on the mechanism and perforation site [37]. Lateral or medial wall perforations (type I) typically require emergent surgery, whereas peri-Vaterian (II) and distal common bile duct injuries (III) are amenable to conservative management. Symptomatic type II patients can benefit from endoclipping [37, 38]. Cases treated conservatively may require follow-up CT after 48–72 h [38]. Isolated retroperitoneal air from insufflation does not require treatment when asymptomatic (type IV) [37, 38].

Perforation in the setting of gastric banding may present as an acute or, more commonly, a chronic complication secondary to transmural band erosion and manifests with free or loculated extraluminal gas outlining the band, fat stranding or a subphrenic abscess [17, 29]. Part of the band may be seen in the gastric lumen [17, 29].

**Fig. 8:**
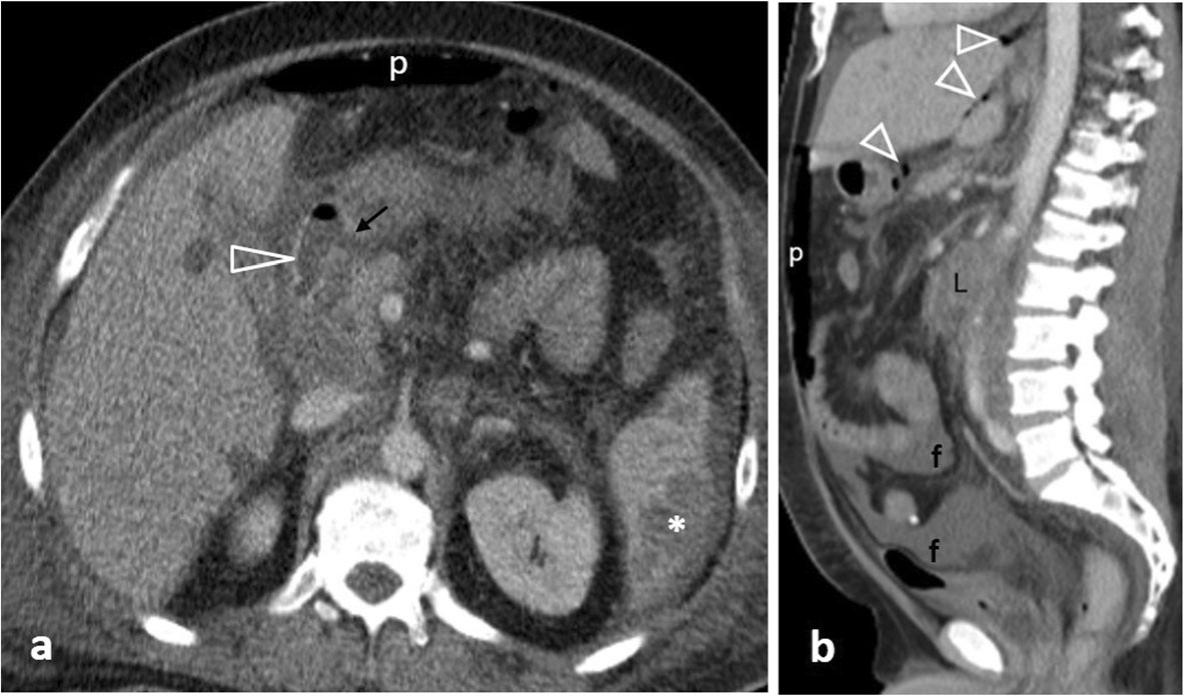
62-year-old patient with perforating gastric carcinoma. **a** Axial and (**b**) sagittal contrast-enhanced images show the enhancing antral mucosa (arrowhead) and a focal discontinuity of mucosal enhancement (arrow) associated with fat stranding, pneumoperitoneum (p), free fluid (f), gas bubbles by the posterior wall of the antrum coursing cranially within the lesser sac (arrowheads), co-existing metastases (*), as well as periaortic lymphadenopathy (L)

Gastric perforation associated with malignancy is rare (0.4–6%), occurring particularly with ulcerated masses, e.g. adenocarcinoma, lymphoma, and large gastrointestinal stromal tumours (GISTs) [2, 29]. Irregular wall thickening, submucosal mass, heaped-up ulcer craters, perivisceral soft-tissue extension, peritoneal spread, lymphadenopathy and distal metastatic disease are highly suggestive of underlying malignancy (Fig. 8) [2, 29, 35].

**Fig. 9:**
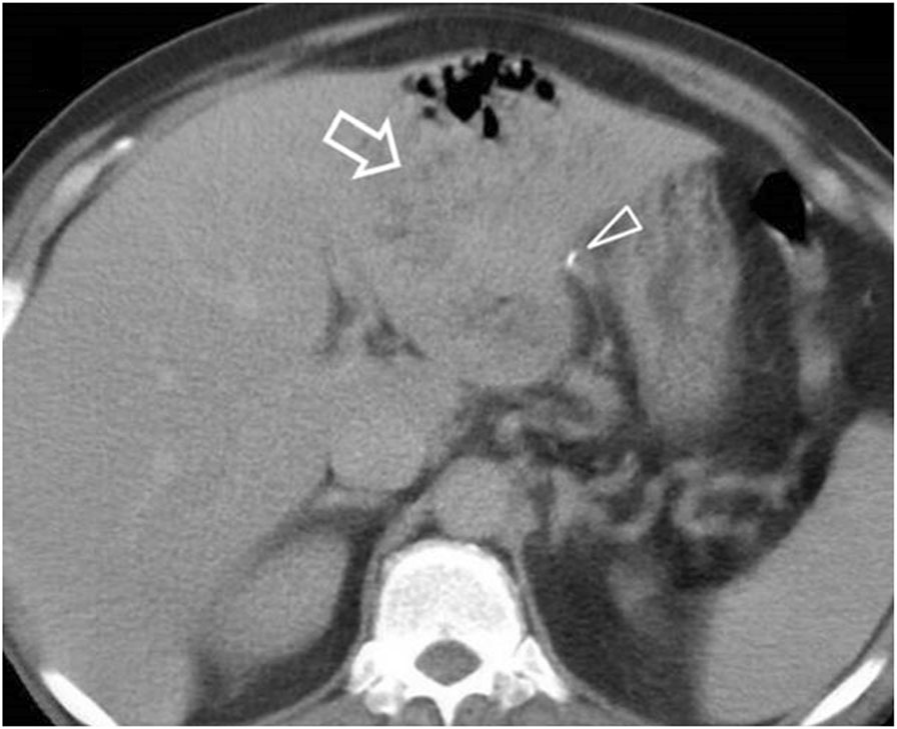
70-year-old patient presenting with fever and right upper quadrant pain for 3 days. Axial contrast-enhanced image demonstrates a gas-containing liver abscess (open arrow) and a hyperdense foreign body in the hepatogastric ligament representing a penetrating fishbone (arrowhead)

Less than 1% of cases of ingested foreign bodies lead to perforation [16, 18]. Some patients may not recall ingesting a foreign body, present with nonspecific symptoms and may be diagnosed months or years later [16]. Patients at higher risk are those with reduced palate sensitivity (use of dentures), alcohol abuse, young children, elderly and mentally handicapped [16, 18]. A foreign body may be radiopaque depending on its composition [18]. Fish bones are the most common culprit throughout the GIT except for the oesophagus and usually result in contained perforations which are sealed off by surrounding omentum and inflammation [16]. Consequently, associated pneumoperitoneum is uncommon [16, 18]. A foreign body may rarely perforate and migrate into an adjacent organ, most commonly the left hemiliver, presenting with fistula and abscess formation (Fig. 9) [ 16,18]. In neglected or chronic cases, where the foreign body gradually erodes through the bowel wall, the resulting inflammatory changes may mimic malignancy. Duodenal perforations in particular may mimic pancreatitis or a pancreatic mass since they may run a longer and relatively asymptomatic course. In these cases, it is essential to identify a hyperdense structure as the offending foreign body [16].

Presenting symptoms, imaging and clinical considerations in relation to site and causes of upper GIT perforation are summarised (Table 1).

**Table 1:** Presenting symptoms, imaging and clinical considerations in relation to site and causes of upper GIT perforation

Site	Presentation	CT findings	Causes	Cause-specific findings	Considerations
Oesophagus	Severe distress, sudden-onset pain, fever, dysphagia, dyspnea, hoarseness, dysphonia, tachycardia, crepitus	Mural defect, pneumomediastinum, free mediastinal contrast, free mediastinal fluid, mural gas, subcutaneous emphysema, wall thickening, mediastinal or cervical fat stranding, pleural effusion	Iatrogenic		History of instrumentation
			Spontaneous		Hyperemesis
			Foreign body	Visible food bolus, impacted foreign body	Investigate for underlying stricture
			Trauma		History of penetrating injury
			Tumour	Massive wall thickening, oesophago-respiratory fistula	History of radiotherapy, instrumentation, stent placement
	Fever, SIRS, shock*				
Gastroduodenal	Acute abdominal pain, guarding, rebound tenderness, non-specific pain (in RP)	Supramesocolic pneumoperitoneum, gas in ligamentum teres, gas in falciform ligament, gas in lesser sac, oral contrast leakage, mural defect, gas in anterior pararenal space (in RP)	PUD	Mucosal hyperenhancement	Helicobacter pylorii infection
				Luminal outpouching	Drugs, stress, tobacco, alcohol abuse
			Trauma	Gas in wound track	History of trauma
				Solid organ injuries	
			Iatrogenic	Gas outlining gastric band, subphrenic abscess	History of instrumentation or history of gastric banding
				Intraluminal band/sutures	
			Tumour	Irregular wall thickening	
				Mucosal/submucosal enhancement	
				Perivisceral soft-tissue extension	
				Peritoneal/nodal spread	
				Metastatic disease	
			Foreign body	Radiopaque structure by a fistula, opacity by a liver abscess	Individuals with reduced palate sensitivity, alcohol abuse, children, elderly, mentally handicapped

*In late stages. *SIRS* systemic inflammatory response syndrome, *RP* retroperitoneal perforation, *IP* intraperitoneal perforation, *RT* right

## Small bowel perforations

Perforation of the jejunum or ileum carries an incidence of 1 in 300–350,000 and accounts for 0.4% cases of acute abdomen [20]. Presentation is nonspecific, consisting of abrupt onset persistent abdominal pain unresponsive to medication which evolves into sepsis and peritonitis if left untreated [2, 5, 39].

Trauma is the leading cause of perforation, followed by closed-loop obstruction and tumours in developed countries and by infection in developing countries (typhoid fever, tuberculosis, HIV and hookworms among others) [5, 20]. Crohn’s disease (CD), ischemia, iatrogenic manipulations, foreign bodies, small bowel diverticulitis and medications (e.g. NSAIDs, potassium chloride) constitute less common causes [2, 5, 20, 27].

Pneumoperitoneum may be absent or too subtle to be detected, noted in approximately half the cases [5, 7, 10, 20, 27]. For this reason, images should be scrutinised for indirect findings including wall thickening, mesenteric fluid and stranding and ancillary findings indicative of the underlying cause such as a mass, an abscess, an incarcerated hernia or a foreign body [5, 12, 20, 27]. Attention must be paid to localised interloop collections of extraluminal fluid or gas, as they can easily be mistaken for intraluminal contents [20].

**Fig. 10:**
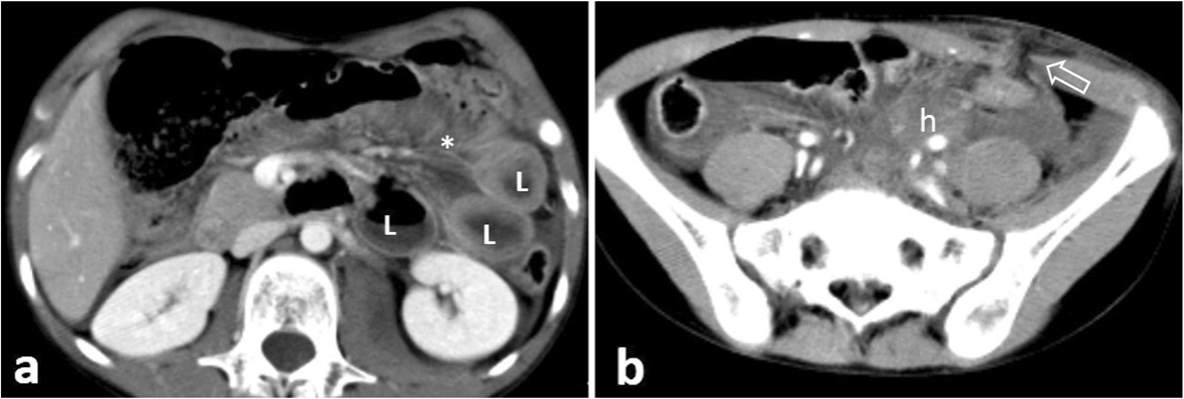
7-year-old patient with jejunal rupture following a handlebar injury. **a**, **b** Axial contrast-enhanced images demonstrate wall thickening of distended jejunal loops (L), mesenteric stranding (*) coalescing into a mesenteric hematoma (h), an unsuspected abdominal wall dehiscence (open arrow) and pneumoperitoneum anterior to the liver (not shown)

**Fig. 11:**
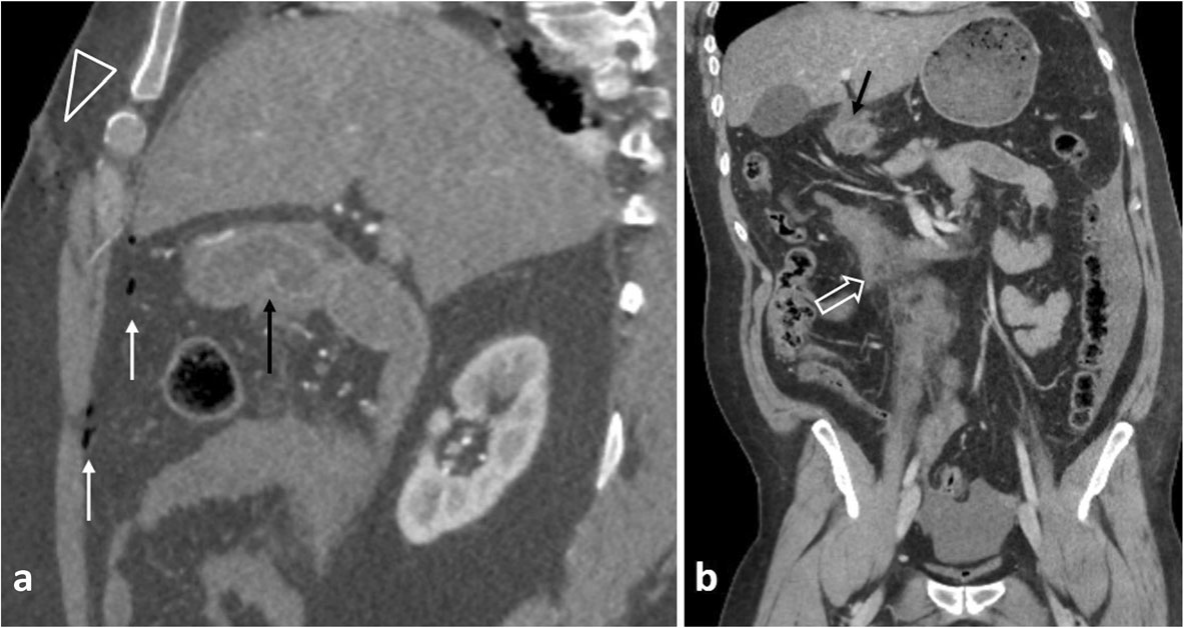
35-year-old patient with duodenal and mesenteric trauma following a gunshot wound. **a** Sagittal and (**b**) coronal contrast-enhanced reformatted images demonstrate gas bubbles along the bullet trajectory (arrowhead), scattered air bubbles (white arrows), periduodenal fat stranding (black arrows) and a mesenteric root hematoma (open white arrow)

Trauma, although uncommon, constitutes the most frequent cause of jejunal/ileal perforation [5]. The small bowel is the third most frequently involved site in abdominal blunt trauma, following the liver and spleen [5]. Since abdominal traumatic lesions are rarely isolated, the abdomen should be scrutinised for coexisting injuries (Fig. 10). CT diagnosis of small bowel blunt injury is challenging because specific signs for bowel injury are frequently absent [20]. The combination of bowel wall thickening and mural discontinuity is the most accurate indicator of bowel injury [14]. Indirect signs which should raise suspicion for occult bowel injury but not necessarily perforation include mesenteric fat stranding and a moderate to large volume of unexplained intraperitoneal fluid in the absence of solid organ injury [2, 14]. Investigation with CT in penetrating injury is controversial as it may significantly delay surgical management [2]. In penetrating injury, free intraperitoneal gas is not diagnostic, as air can be introduced into the peritoneal cavity by the entry wound [14] (Fig. 11). In this setting, demonstrating a wound track that extends to an injured intestinal segment is the most sensitive finding [2, 14].

**Fig. 12:**
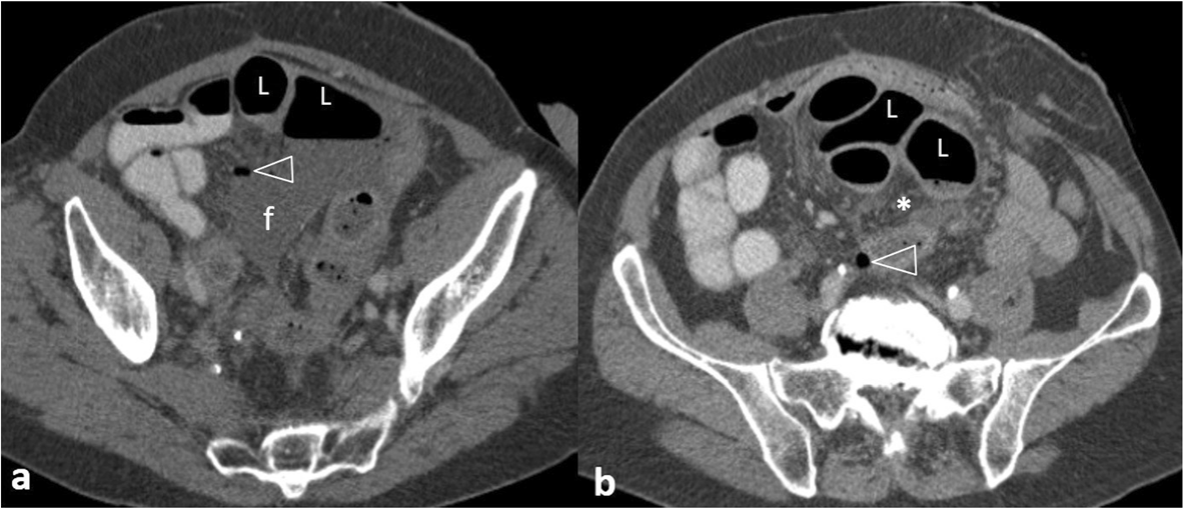
72-year-old patient with small bowel perforation following closed loop strangulation. **a, b** Axial contrast-enhanced images following oral contrast administration demonstrate distended air-filled poorly enhancing bowel loops (L) within an internal hernia’s sac. Note free gas (arrowheads), localised fluid (f), fat stranding and mesenteric vessel congestion (*)

**Fig. 13:**
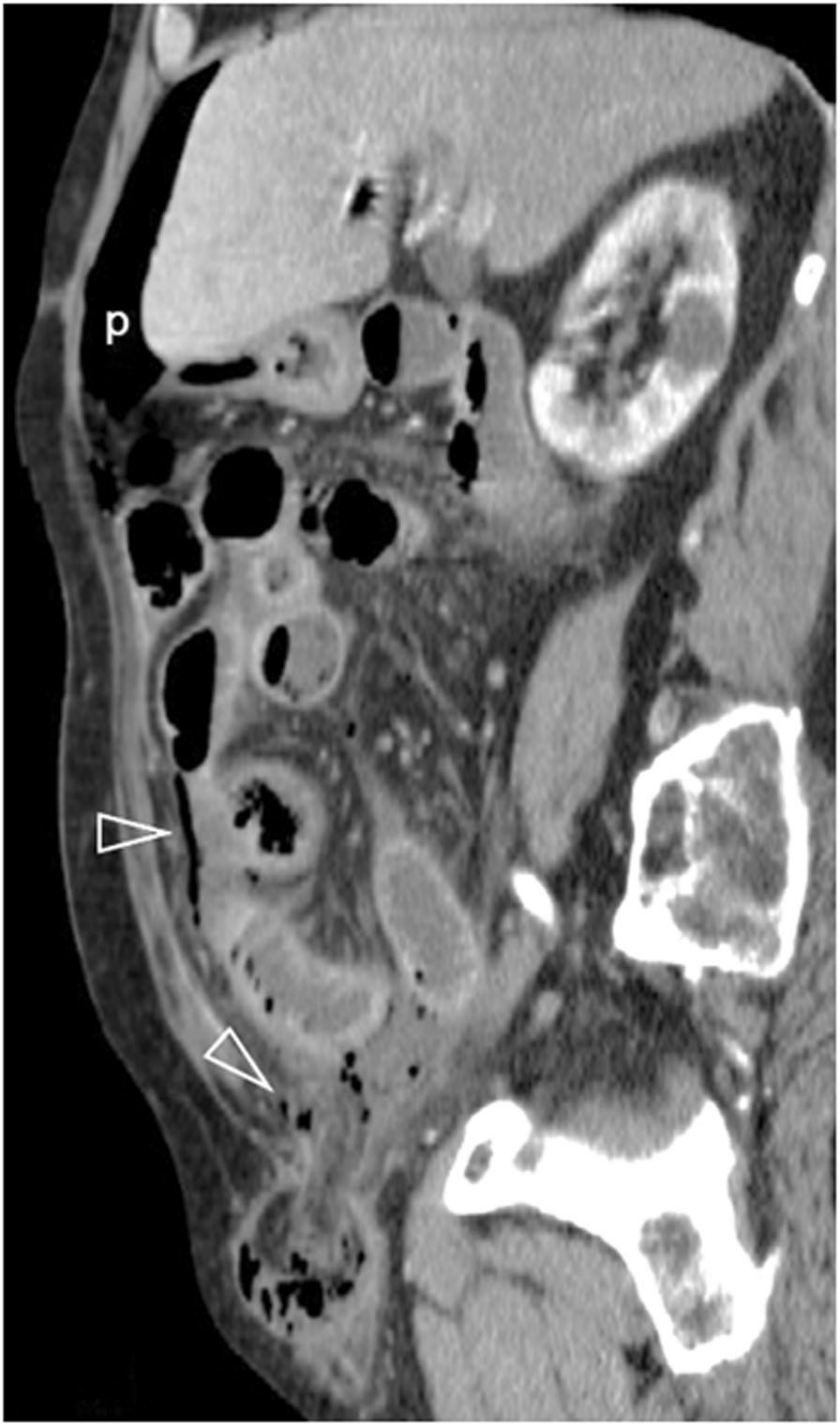
60-year-old patient with a right incarcerated inguinal hernia. Sagittal oblique contrast-enhanced image depicts a poorly enhancing ileal loop inside the hernia, free intraperitoneal gas bubbles (open arrowheads) and periportal gas coalescing to pneumoperitoneum (p)

Perforation secondary to ischemia can result from either strangulated bowel obstruction (Figs. 12 and 13) or from a primary vascular event (large vessel occlusion, venous outflow obstruction, or vasculitis) [20]. Ischemia can additionally result from marked and prolonged hypotension secondary to sepsis, congestive heart failure, acute myocardial infarction and hypovolemic shock. CT changes suggestive of underlying ischemia mainly depend on the aetiology as well as the duration and extent of the ischemic attack and include perfusion abnormalities, ranging from bowel wall hyperenhancement to reduced or absent wall enhancement, segmental bowel wall thickening, localised fluid/fat stranding and emboli or thrombi in mesenteric vessels. Pneumatosis intestinalis and portomesenteric gas in the setting of mesenteric ischemia indicate transmural infarction [2, 5, 20].

**Fig. 14:**
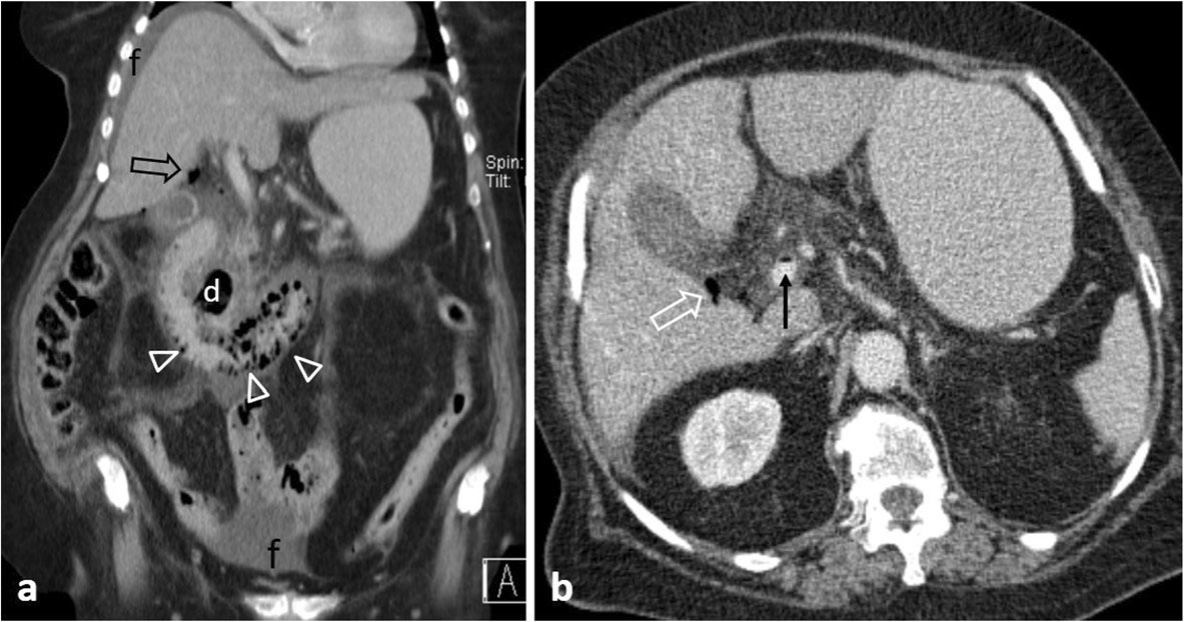
75-year-old patient with vasculitis. **a** Coronal contrast-enhanced illustrates pneumatosis intestinalis, and poor enhancement of the horizontal and ascending duodenum (arrowheads) and adjacent fat stranding. Note an incidental diverticulum (d) at the 2nd part of the duodenum, moderate amount of free fluid (f) and gas bubbles (open arrows) at the porta hepatis. **b** Axial CT image demonstrates gas in the superior mesenteric vein (black arrow) with adjacent fat stranding

Small vessel vasculitis is a rare cause of intestinal perforation. Diagnosis is based on indirect signs including a non-vascular territory distribution of multiple and occasionally discontinuous ischemic bowel segments [2, 40]. Ischemic involvement of the duodenum is nearly always indicative of vasculitis (Fig. 14) [40].

**Fig. 15:**
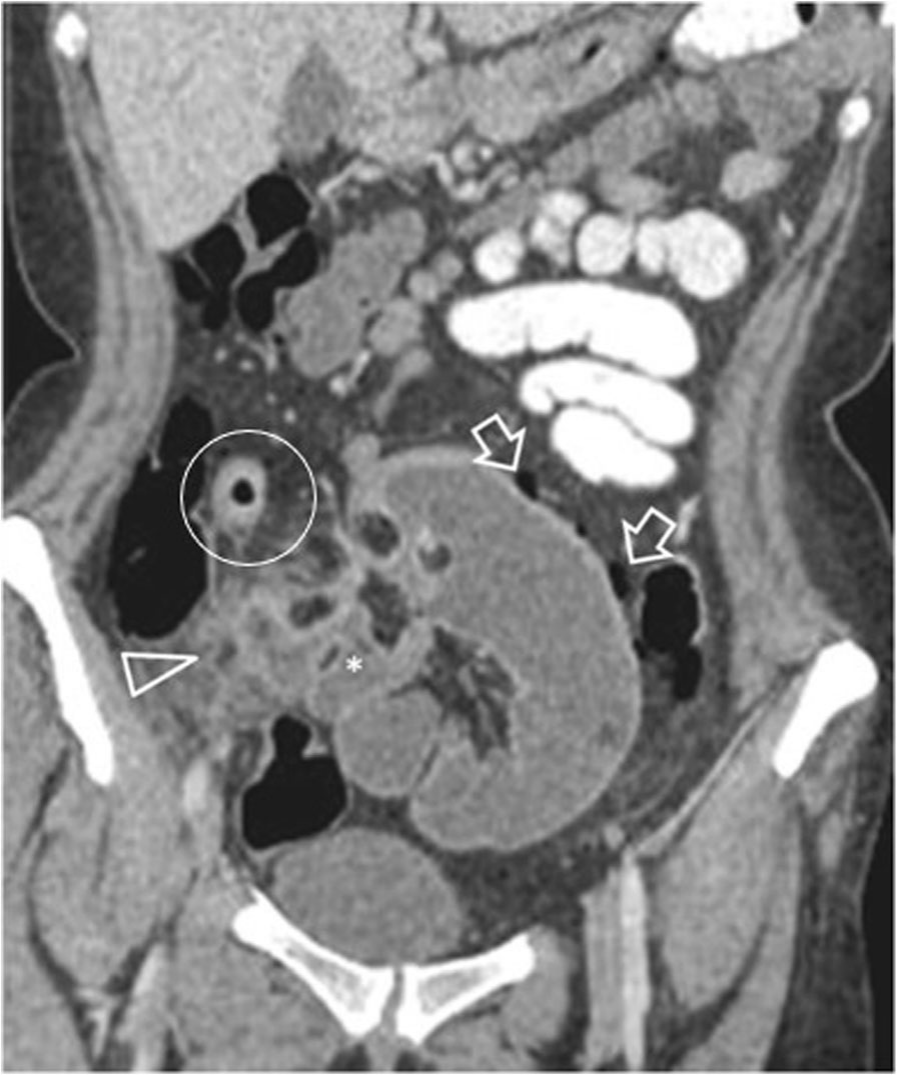
40-year-old patient with ileal perforation and unknown Crohn’s disease. Contrast-enhanced image shows fluid-filled fistulous ileoileal tracts (*), adjacent mesenteric phlegmon (arrowhead) and pockets of intraperitoneal free gas (arrows). Note mural thickening with stratification and mucosal hyperenhancement of the terminal ileum (within circle) compatible with active inflammation

Approximately 75–80% of patients with Crohn’s require surgery within the first 5–20 years from diagnosis due to the development of stricturing or penetrating disease [41, 42]. Transmural Crohn’s may lead to contained perforation due to the presence of adhesions between adjacent structures or bowel loops [20, 27]. Subsequent phlegmon and abscess formation with localised peritonitis may develop [14]. Extraluminal complications of penetrating CD also include sinus tracts and/or fistulas between bowel loops and between bowel and other visceral organs. Free perforation is a rare life-threatening complication that occurs in 1–3% of cases [2, 14, 20] (Fig. 15). Additional findings like discontinuous and/or long segment bowel wall thickening, engorgement of vasa recta adjacent to an inflamed bowel loop and mural stratification characterise active disease, and may suggest the aetiology in otherwise undiagnosed CD [2, 14]. Inflammatory stranding in the small bowel mesentery adjacent to a thick-walled segment of bowel is not specific for perforation in patients with CD [27].

Perforation secondary to small bowel tumours occurs more often in primary malignant lymphoma especially when treated with chemotherapy and steroids, in post-transplant lymphoproliferative disorder and following radiation therapy [2, 14, 20], although adenocarcinomas, GISTs and metastases can also perforate [20]. Circumferential bowel wall thickening with luminal aneurysmal dilatation is highly suggestive of lymphoma [2]. Additional findings suggesting GIT lymphoma are multifocal bowel involvement, lymphadenopathy and hepatosplenomegaly [2, 43]. GISTs rarely perforate and in this setting usually present with heterogeneous attenuation and a lamellated pattern reflecting areas of haemorrhage or necrotic degeneration [2]. Since ascites is uncommon in GISTs, such an associated finding should prompt thorough inspection for tumour rupture and/or metastatic spread [2].

**Fig. 16:**
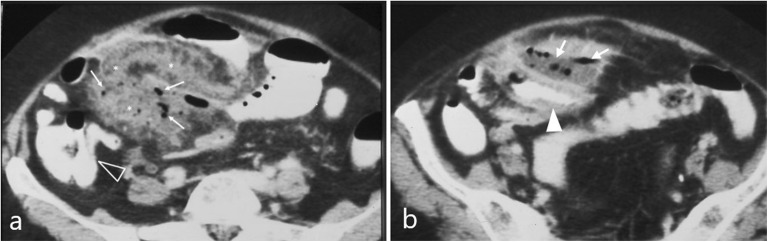
82-year-old patient with perforated jejunal diverticulum. **a**, **b** Axial contrast-enhanced images demonstrate extensive mesenteric fat stranding (*) and free gas bubbles (arrows) relatively contained in a fluid collection around a contrast-filled jejunal loop with wall thickening (white arrowhead). Note the normal appearance of the terminal ileum and ileocaecal valve (arrowhead) that makes the diagnosis of inflammatory bowel disease unlikely

Small bowel diverticula occur in 0.06–2.3% of the population and rarely perforate [20] (Fig. 16). Meckel’s diverticulum is located at the antimesenteric side wall of the distal ileum, most commonly containing gastric mucosa (62%), and can be complicated by bleeding and, rarely, perforation [27, 44]. Reformatted images can better illustrate the relationship of diverticula to the lumen of the bowel and suggest them as the perforation site [20].

**Fig. 17:**
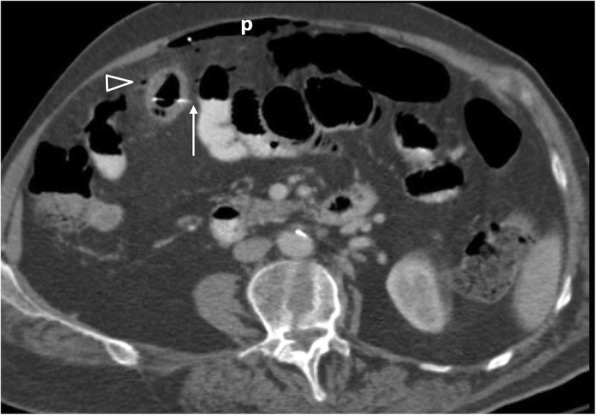
78-year-old patient. Contrast-enhanced axial image demonstrates a linear hyperdense foreign body partly protruding through the ileal wall, representing a fishbone (arrow). Note the concentric wall thickening of the affected loop, few adjacent gas bubbles (arrowhead) and pneumoperitoneum (p)

Ingested foreign bodies may rarely cause small bowel perforation [27]. Common sites include narrowed or angulated portions of the GIT, such as the ileocaecal area [14, 16, 18, 27]. As with gastroduodenal perforations, pneumoperitoneum is minimal if any, since the foreign body is gradually impacted and is walled-off by omentum and inflammatory changes [2, 16, 20] (Fig. 16). Identifying a partially extraluminal foreign body confirms the diagnosis, and it is more easily visualised in bone window settings (Fig. 17) [2, 14]. The foreign body may be identified distal to the perforation site, since it is possible to cause a perforation and then move distally within the bowel lumen [20].

Iatrogenic perforation following open or laparoscopic surgery most commonly involves the small bowel and carries high morbidity and mortality, especially if not recognised intraoperatively [2, 20]. Anastomotic leakage usually occurs within the first postoperative week [10]. Endoscopic procedures, misplaced percutaneous drainage catheters, radiation-induced injury and paracentesis can also result in small bowel injury and subsequent perforation [20]. Intraperitoneal free gas is difficult to interpret since it is normally expected post-laparotomy for up to 2 weeks and approximately for up to 3 days following laparoscopic procedures [7]. Oral contrast may be useful in this setting, as contrast leakage with an intact anastomotic site suggests the diagnosis of accidental iatrogenic bowel injury [2, 10, 14]. Persistent or progressively increasing free air and/or ascites should raise concern for perforation or anastomotic leakage [8, 10].

## Appendiceal perforation

Appendiceal perforation results from obstruction due to an appendicolith in the setting of appendicitis or may rarely be associated with an underlying tumour or mucocele [8, 27]. Appendicitis is rarely caused by obstructing fruit seeds, vegetables, lymphoid hyperplasia, intestinal worms (Ascaris), malignancy, and foreign bodies [45]. Delay in presentation is strongly associated with perforation in the setting of appendicitis [45].

The role of CT in detecting perforating appendicitis at an early stage or appendiceal micro-perforations is controversial, especially in young patients [2]. In neglected/complicated cases, elderly patients and possible dual pathology, CT has an established role.

**Fig. 18:**
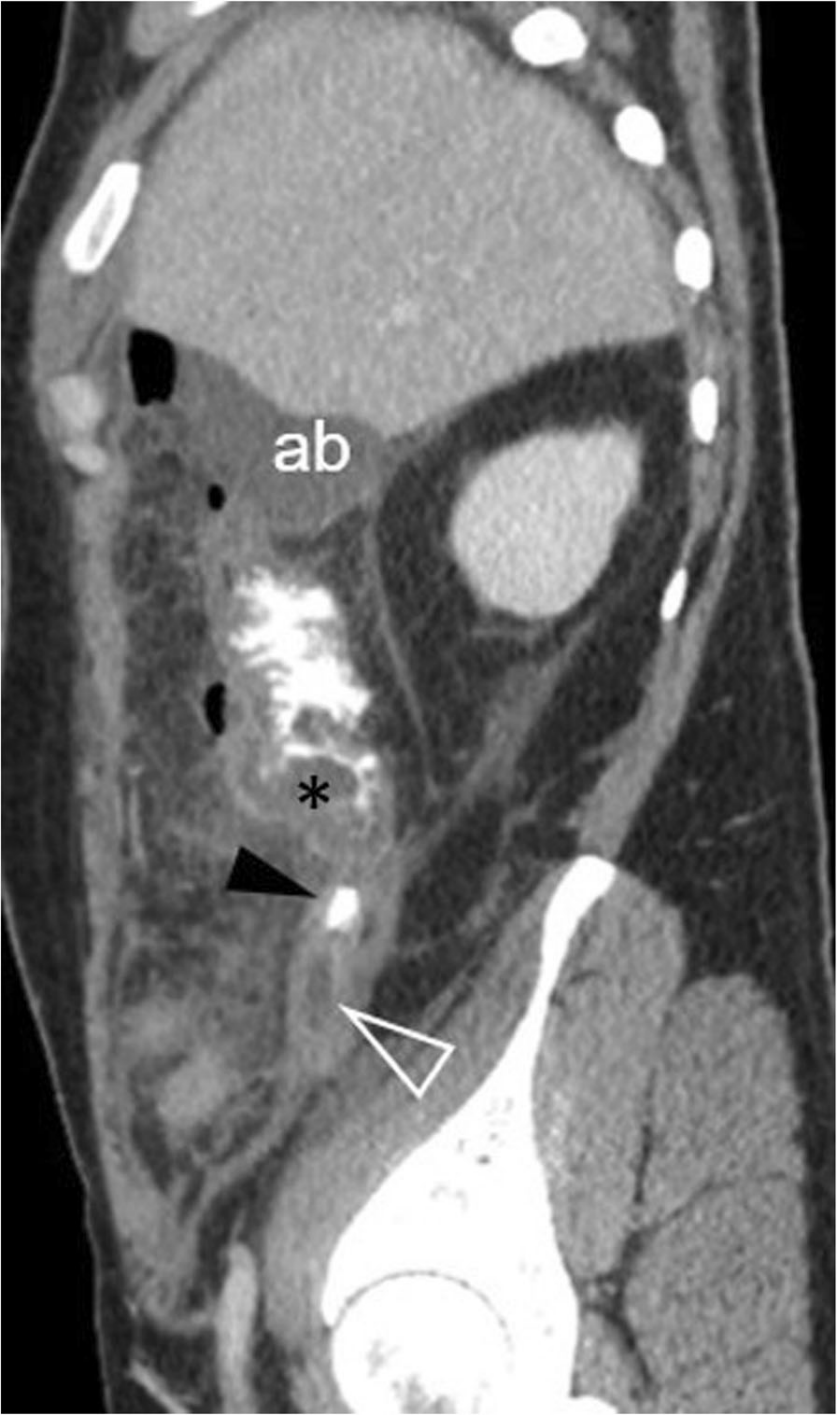
42-year-old patient with appendiceal perforation. Contrast-enhanced parasagittal reformatted image shows a dilated appendix (open arrowhead) containing an obstructive appendicolith (black arrowhead), reactive wall thickening of the ascending colon (*), surrounding fat stranding, as well as an abscess (ab) containing air-fluid levels

The presence of extraluminal gas, which is usually minimal (< 2 mL) or absent, an appendiceal wall defect, periappendiceal abscess, and extraluminal appendicolith are highly suggestive of perforation [2, 7, 8, 10, 27] (Fig. 18).

**Fig. 19:**
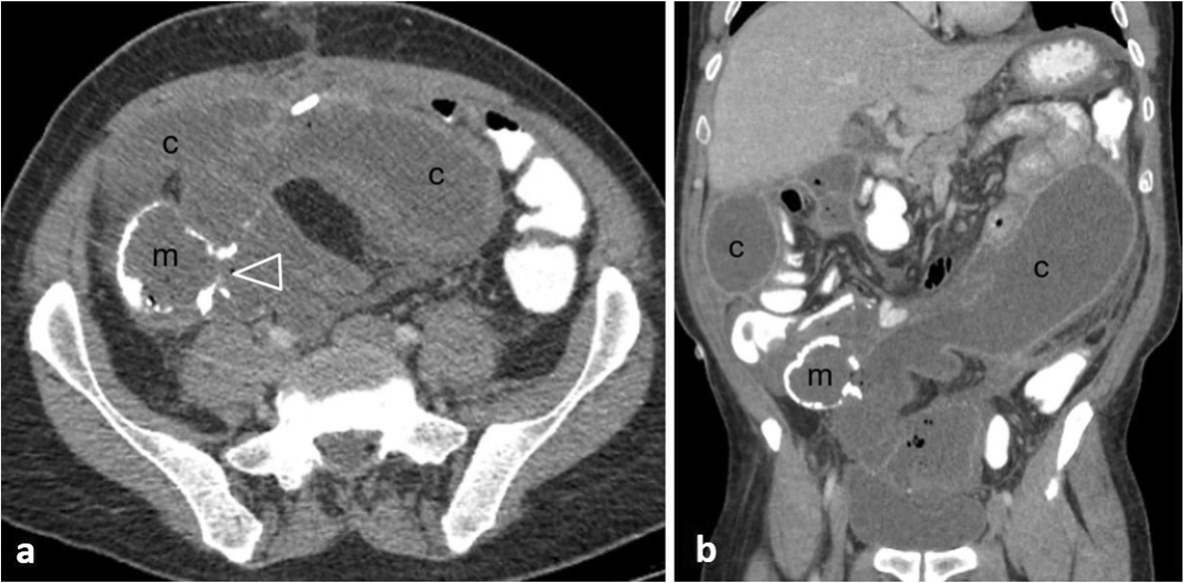
65-year-old patient with a ruptured appendiceal mucinous neoplasm. **a** Axial and (**b**) coronal contrast-enhanced image shows a hypodense mass (m), surrounded by curvilinear calcifications. Note the extensive enhancing encapsulated fluid collections (c), typical of pseudomyxoma peritonei, arising from a focal discontinuity (arrowhead) of the calcified wall of the tumour

Cystic dilatation of the appendix with a luminal diameter greater than 1.3 cm, along with the presence of mural calcifications, suggests a mucocele or a mucinous neoplasm [2, 46]. Secondary perforation, caused by an appendiceal mucinous neoplasm, leads to pseudomyxoma peritonei [2] (Fig. 19).

Presenting symptoms, imaging and clinical considerations in relation to site and causes of small bowel and appendiceal perforation are summarised (Table 2).

**Table 2:** Presenting symptoms, imaging and clinical considerations in relation to site and causes of small bowel and appendiceal perforation

Site	Presentation	CT findings	Causes	Cause specific findings	Considerations
Small bowel	Non-specific, abrupt sudden-onset pain, vomiting, anorexia, nausea, sepsis, peritonitis	IP gas (minimal/absent), oral contrast leakage, mural defect, wall thickening, poor/inhomogeneous mural enhancement, mesenteric fluid, fat stranding, extraluminal faecal material	Trauma	Pneumoperitoneum not diagnostic in penetrating trauma, wound track extending to intestinal segment.	History of trauma
			IIschemia	Decreased/absent bowel enhancement, pneumatosis intestinalis, gas/thrombi within mesenteric/portal vessels	Mechanical obstruction, large vessel occlusion, venous outflow obstruction, vasculitis, sepsis, congestive heart failure, acute MI, hypovolemic shock
			Inflammatory	Phlegmon/abscess formation	Typhoid fever, HIV, tuberculosis, hookworms
			IBD	Phlegmon/abscess formation, lengthy bowel wall thickenning, sinus tracts, fistulas	Crohn's disease
			Tumour	Circumferential wall thickening aneurysmal luminal dilatation multifocal bowel involvement lymphadenopathy hepatosplenomegaly heterogeneous mass	Commonly lymphoma adenocarcinoma malignant GISTs metastases
			Diverticulae	Inflamed diverticulum	Meckel's diverticulum
			Foreign body	Foreign body, may be located distal to perforation site	Common in ileocaecal area Avoid oral contrast
			Iatrogenic	persistent/progressively increasing free gas and/or ascites, oral contrast leakage	Laparoscopic surgery, anastomotic leakage, endoscopic procedures. Pneumoperitoneum normal < 2 weeks post laparoscopy
Appendix	Long standing abdominal pain, fever, muscle guarding	Periappendiceal/IP gas, appendiceal wall defect, phlegmon/abscess, fat stranding, free fluid	Inflammation	Extraluminal appendicolith	
			Tumour	Luminal diameter > 1.3 cm, mural calcifications, pseudomyxoma peritonei, mass, enhancing wall nodularity	

*IP* intraperitoneal perforation, *MI* myocardial infarction, *HIV* human immunodeficiency virus, *IBD* inflammatory bowel disease, *GIST* gastrointestinal stromal tumour

## Colorectal perforation

Colorectal perforation carries the highest complication rate (55%) compared with other GIT perforation sites [47]. This is unsurprising considering the bacterial content of the large bowel that evokes bacterial peritonitis [1, 4]. Perforations contained in retroperitoneal spaces usually present with subtle symptomatology [6, 27].

Malignancy is the commonest cause, accounting for 36% of perforations [48]. Perforation may also be iatrogenic (20%), diverticula-related (19%) and less commonly due to trauma, foreign body ingestion, faecal impaction, ischemia, inflammatory bowel disease, endometriosis, connective tissue disease, radiotherapy, drugs and spontaneous [4, 6, 7, 10, 11, 49]. The site of perforation is often linked with its cause [4, 8, 10]; therefore, neoplastic, spontaneous, diverticular (in western countries), blunt trauma and ischemic perforations commonly occur on the left side of colon, whereas inflammatory bowel disease, diverticulitis (in eastern countries) and penetrating trauma perforations tend to be right-sided. The rectosigmoid is most commonly affected in iatrogenic manipulations and foreign bodies [4, 6]. The cecum may perforate when it reaches 12–14 cm in diameter as a result of a rapid increase in intraluminal pressure in bowel obstruction with a competent ileocaecal valve, toxic megacolon, stercoral colitis and acute colonic pseudo-obstruction [4, 8, 11]. Extraluminal gas limited in the pelvis or within retroperitoneal compartments suggests colorectal perforation with the exception of right-sided retropneumoperitoneum which may also result from duodenal perforation.

The accuracy of MDCT in predicting the perforation site is lower in colorectal perforations compared with upper GIT perforations [9]. Direct findings include extraluminal gas and oral/rectal contrast, as well as wall discontinuity, faecal material looking like a “dirty” mass or foreign bodies protruding through the colonic wall or lying free within the abdominal cavity [4, 8, 11, 27, 50]. Associated findings include bowel wall thickening, pericolonic fat stranding, free fluid, abnormal wall enhancement, abscess, and the presence of a localised inflammatory mass adjacent to the colon [4, 8, 11, 27].

Free gas may be massive, especially if there is coexistent obstruction or following colonoscopy [4, 27]. Perforation of intraperitoneal colon, i.e. of cecum, transverse, sigmoid and upper 2/3 of the rectum, leads to pneumoperitoneum [7]. Rupture of the retroperitoneal ascending and descending colon results in pneumoretroperitoneum at the right and left anterior pararenal spaces respectively [2, 7, 10, 11]. Posterior rectal perforations can dissect superiorly and cause bilateral pneumoretroperitoneum [10, 11]. In the setting of diverticulitis or malignancy without mechanical obstruction, perforation is often associated with small volume pneumoperitoneum by the perforation site [8, 10]. Free gas present only in the pelvis favours colonic rather than small bowel perforation [2, 6, 11, 27].

**Fig. 20:**
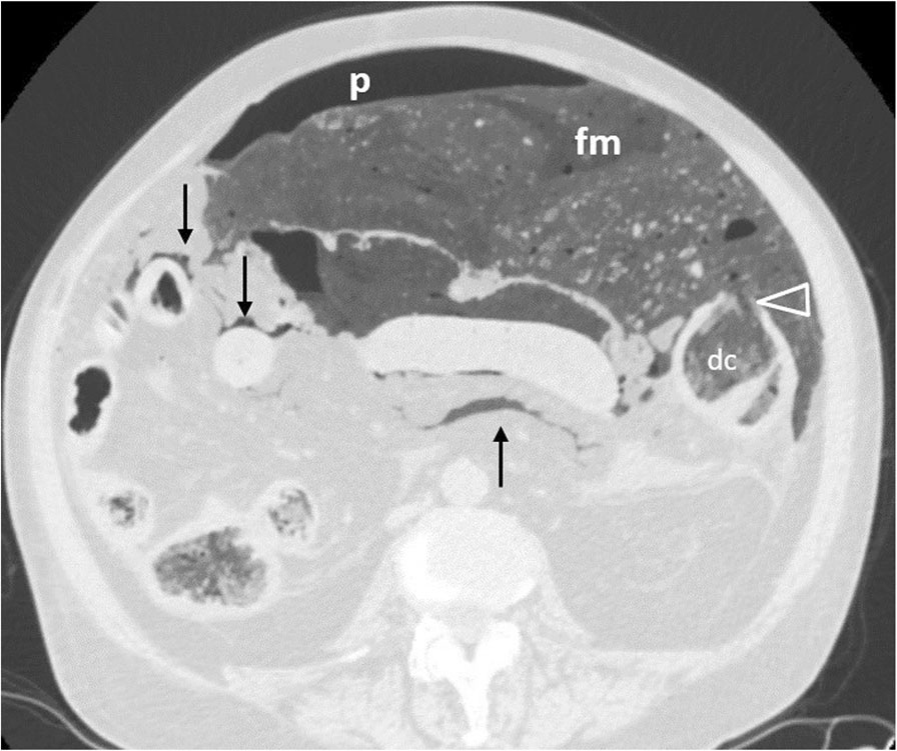
75-year-old patient with perforation secondary to obstructive ileus in the setting of rectal cancer (not shown). Axial image (lung window) shows leaked faecal material (fm), abutting the mesenteric root and surrounding ileal loops (arrows), pneumoperitoneum (p), and the perforation site (arrowhead) as a wall discontinuity of the descending colon (dc)

**Fig. 21:**
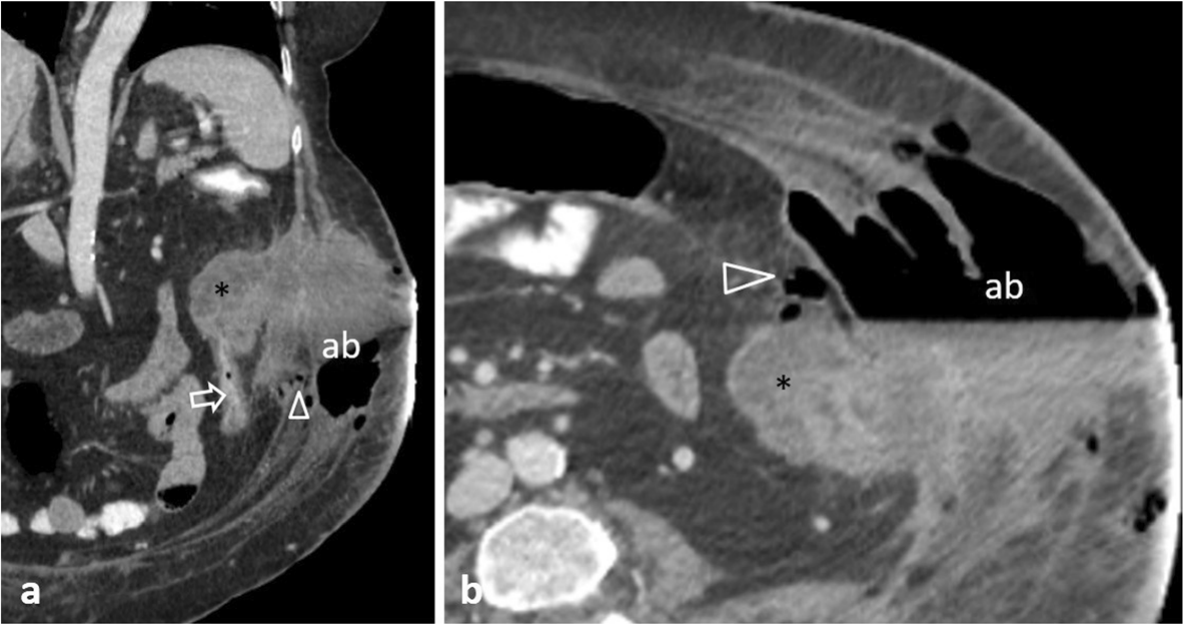
62-year-old patient with colonic rupture into the abdominal wall. **a** Coronal and (**b**) axial contrast-enhanced images illustrate a soft tissue mass (*) arising from the descending colon (arrow), extending into an abscess (ab) containing air-fluid level. Note the extraperitoneal gas bubbles (arrowheads) abutting the muscular fascias

**Fig. 22:**
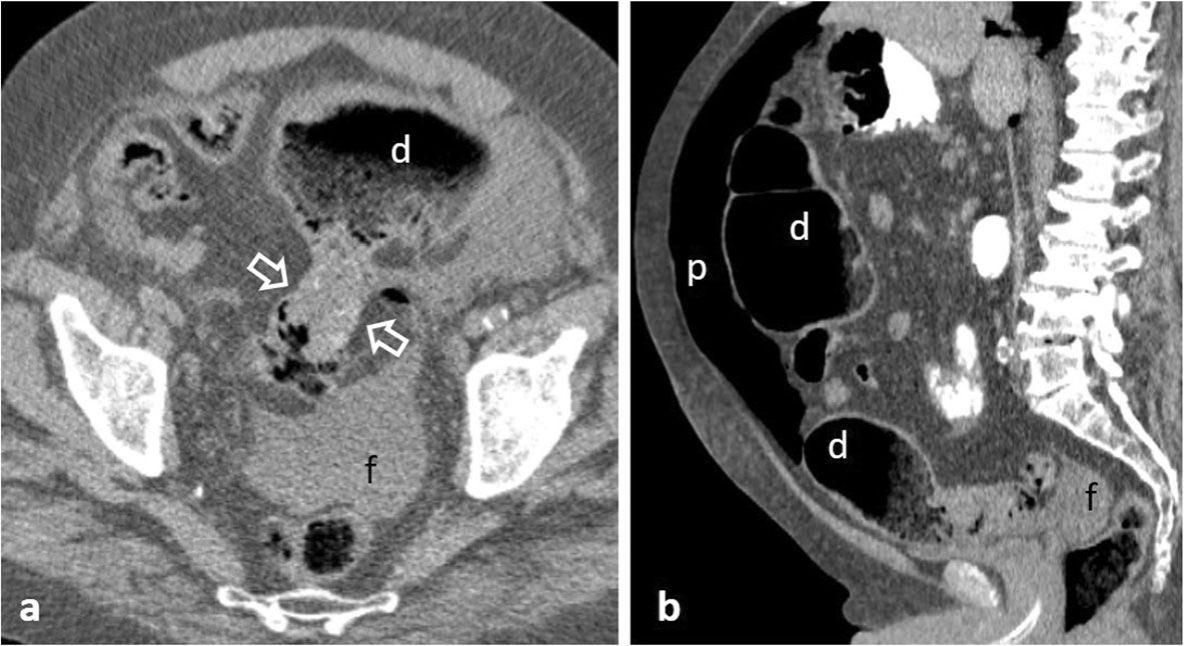
75-year-old patient with obstructive sigmoid carcinoma. **a** Axial and (**b**) sagittal non-enhanced images show concentric narrowing of the sigmoid lumen (between arrows), prestenotic colonic dilatation (d), massive pneumoperitoneum (p) and free fluid (f)

Perforation complicates 2.6–10% of colon cancers, occurring most commonly in the sigmoid colon (47.3%) and cecum (24.8%) [2, 6, 11, 49, 51]. Two mechanisms are recognised [2, 4, 6, 11, 49]: the first consists of tumour necrosis and subsequent perforation at the tumour site. The second occurs proximal to the malignancy, as a result of bowel distension secondary to obstruction, frequently occurs at the cecum and usually leads to massive pneumoperitoneum (Fig. 20) or pneumoretroperitoneum. Perforation secondary to tumour necrosis usually causes a small amount of free gas [11]. Rarely, the colon perforates into the abdominal wall and patients may present with extensive cellulitis (Fig. 21). Differentiation between neoplastic and inflammatory causes is challenging; however, a mural wall thickness greater than 1.39 cm, the presence of irregular wall configuration, adjacent lymphadenopathy and metastatic disease favour malignancy since perforation is usually associated with an advanced tumour stage [51] (Fig. 22).

**Fig. 23:**
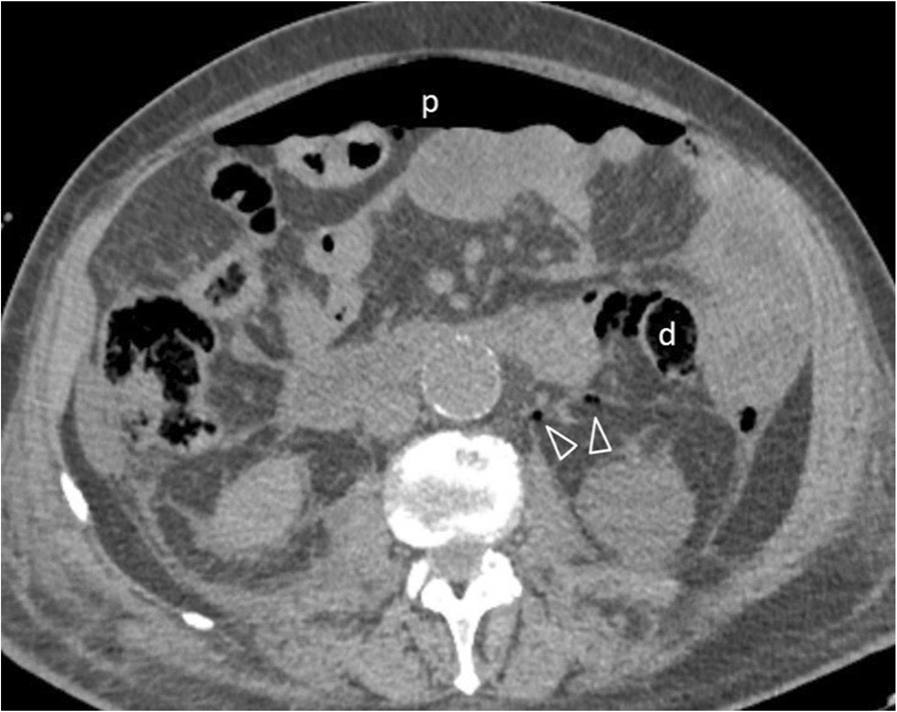
73-year-old patient with perforated diverticulitis. Gas bubbles coalesce towards the descending colon (d), associated with pneumoperitoneum (p) and sparse retroperitoneum (arrowheads)

Perforation secondary to diverticulitis is estimated at 4/100,000 population per year predominantly involving the sigmoid colon [11, 52]. In addition to typical features of diverticulitis, extraluminal air/contrast and pericolic abscesses can be found in localised perforation, while extensive peritonitis occurs in free perforation into the peritoneal cavity (Fig. 23) [6]. Less frequently, perforated sigmoid diverticulitis follows an extraperitoneal route [11, 27]. Purely retroperitoneal forms are exceedingly rare [53].

Iatrogenic perforations are most likely to occur in diseased rather than in healthy bowel [4]. They comprise 20% of colorectal perforations, usually occurring post colonoscopy and most commonly involving the sigmoid (40.7%), followed by the rectum and cecum [4, 6, 11]. Barotrauma from pneumatic distention causes right-sided colonic tears, whereas left-sided rupture results from mechanical trauma from the endoscope on the antimesenteric bowel wall [2]. Polypectomy slightly increases the perforation rate, occurring at the site of the excised polyp [4, 6]. Post-polypectomy syndrome must be considered in these cases, as it clinically mimics perforation and can present with focal mural thickening, pericolonic fluid and fat stranding without free gas [2]. Other iatrogenic causes of perforation include anastomotic leakage, trauma from laparoscopic or robotic manipulations, electrocautery, and percutaneous/endoscopic procedures in the area, such as abscess drainage, paracentesis, colonoscopic stent placement and rarely following a cleansing enema [4, 6]. Anastomotic leakage commonly manifests 5–7 days postoperatively [11]. In perforation from a colonic stent, the visualisation of the stent extending through the site of colonic wall disruption confirms the diagnosis [2].

**Fig. 24:**
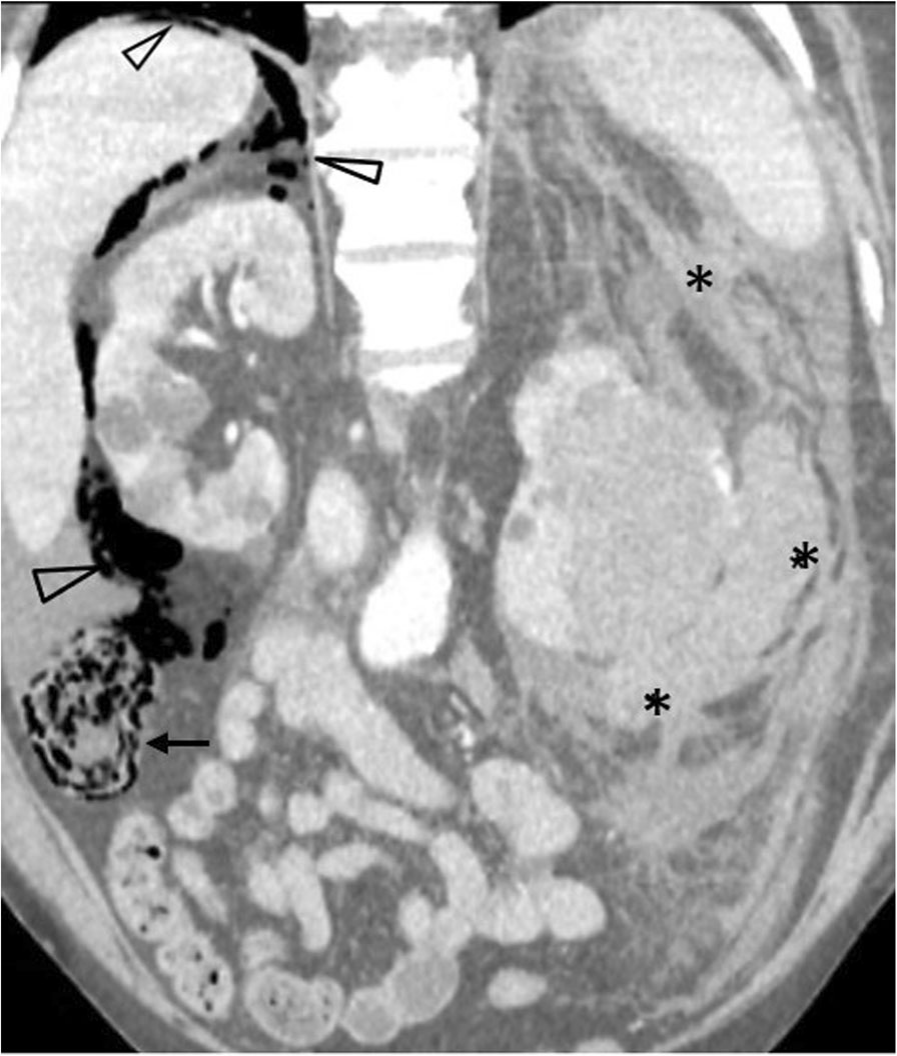
82-year-old oncology patient presenting with spontaneous perforation of the ascending colon. Coronal oblique contrast-enhanced image (modified soft tissue window) depicts pneumatosis intestinalis (arrow) and free gas extending to the right pararenal space and to the bare area of the liver (arrowheads) consistent with retropneumoperitoneum. A left retroperitoneal haemorrhage is noted as an independent finding (*)

Perforation is an uncommon complication of many chemotherapeutic regimens, including taxanes, cytarabine, CHOP (cyclophosphamide, hydroxydaunorubicin, vincristine, prednisolone), axitinid, fluorouracil, cisplatin, mitomycin C, IL-2, ipilimumab, rituximab, erlotinib and bevacizumab and rarely in kayexalate use [11, 54–56]. Bevacizumab specifically has been implicated as the most common cause of drug-induced perforation, most frequently in the setting of metastatic colorectal cancer and epithelial ovarian cancer [33, 55]. Perforation typically occurs within 6 months from treatment onset (Fig. 24). Corticosteroids, NSAIDs and opioids have been associated with perforation in the setting of sigmoid diverticulitis [54]. Corticosteroids in high doses can mask signs of acute abdomen and significantly delay diagnosis. Chronic radiation enteritis can present with strictures, fistulae formation, abscess formation, perforation, and bleeding, as early as 2 months or as late as 30 years following radiation therapy [57].

**Fig. 25:**
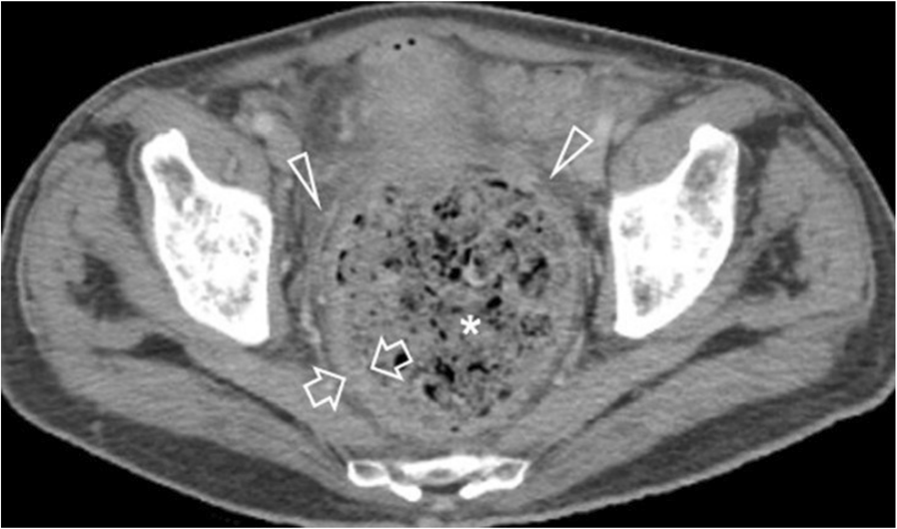
87-year-old patient with stercoral colitis and imminent stercoral perforation. Axial contrast-enhanced image shows a giant faecaloma (*), compressing a thickened rectal wall (between arrows), associated with perirectal fat stranding (arrowheads)

Faecal impaction and stercoral colitis are faecal retention conditions, occurring in the elderly, in chronic constipation, in scleroderma and in bedridden patients [6]. Faecal impaction is characterised by colonic distention caused by a faecaloma (a localised hard faecal mass) in the absence of bowel wall thickening [11, 50]. If the intraluminal pressure increases sufficiently, bowel wall perfusion abnormalities may develop and faecal impaction can progress to stercoral colitis [11], a rare life-threatening condition leading to pressure necrosis and perforation [6, 8, 11, 13, 50]. Stercoral colitis should be suspected when colon dilatation (> 6 cm), bowel wall thickening (> 3 mm) and fat stranding are present at a site of faecal impaction (Fig. 25) [58]. Stercoral perforation is a challenging diagnosis, accounts for 3.2% of all colonic perforations and most commonly involves the apex of the sigmoid colon (50%), the antimesenteric border of the rectosigmoid junction (24%) and the anterior rectum proximal to the peritoneal reflection (7%) [6, 11, 13, 50]. The mortality rate reaches 57%. Stercoral-related ulcers are typically multiple and located on the antimesenteric border of the bowel due to its watershed vascularity and its sensitivity to mechanical constraint [11, 50]. The bowel and rectum distal to a faecaloma should be scrutinised for an obstructing mass as the underlying cause of faecal stasis. In scleroderma patients, the smooth muscle of the bowel or vascular wall may be replaced by collagen which can result in colonic perforation by either stercoliths or vascular impairment [6].

Colorectal foreign bodies, either ingested or following retrograde insertion, are diagnosed with plain radiography [2]. In the presence of peritoneal symptoms or if the foreign body cannot be removed transanally, CT reveals its exact location and assesses possible complications [2, 4, 13]. Bowel wall disruption and extraluminal position of the object are direct findings of perforation, whereas free gas is uncommon as the perforation site is usually “sealed-off” [2, 6, 11, 16, 18]. Occasionally a colovesical fistula or inflammatory mass is encountered [16].

Perforation secondary to ischemic colitis predominantly results from non-occlusive disease (low-flow state) rather than complete vascular occlusion [11]. Low-flow states particularly affect watershed areas, such as Griffith’s and Sudeck’s critical points, whereas arterial occlusion affects whole vascular territories [11, 17]. These patients may initially present with mild abdominal pain and tenderness and develop bloody diarrhoea within 24 h from symptom onset. Pneumatosis intestinalis in the setting of ischemia is associated with poor prognosis [17]. Poor or no mural enhancement is suggestive of ischemia while free air is pathognomonic of transmural necrosis [59].

Acute colonic pseudo-obstruction (Ogilvie’s syndrome) usually affects patients with significant comorbidities or postoperatively [11]. CT findings comprise marked diffuse dilatation of colonic and rectal lumen without a transition point to collapsed bowel [11]. Although perforation is a rare complication (1–3%), carrying a 50–71% mortality rate, the risk increases up to 23% when caecal diameter is > 14 cm [11].

In Ehler-Danlos syndrome, spontaneous perforation is the most common GIT complication, affecting most commonly the sigmoid colon [60]. Perforation usually occurs early in the disease (mean age of presentation 24–26 years), usually precedes arterial or solid organ rupture and can be the first symptom in a young patient otherwise undiagnosed with Ehler-Danlos syndrome [60, 61].

Perforation from blunt trauma is uncommon, being reported in approximately 0.5% of all major blunt traumas [4, 13], more commonly in the sigmoid, right colon and cecum [4]. Transverse colon injury may coexist with pancreatic and/or duodenal injury [4]. Oral contrast administration, when not contraindicated in the emergency setting, can increase the detection rate of bowel wall thickening and small mesenteric hematomas [13]. Free fluid typically forms polygonal-shaped collections among loops and within mesenteric folds [4]. Traumatic perforation of a retroperitoneal segment tends to remain localised adjacent to the injury site [4].

Colonic perforation in inflammatory bowel disease is rare, with free perforation occurring in about 3% of patients with Crohn’s, less common compared with sealed-off perforations [6]. Free perforation from ulcerative colitis occurs in about 2% of patients and is associated with toxic megacolon [6].

Presenting symptoms, imaging and clinical considerations in relation to site and causes of colorectal perforation are summarised (Table 3).

**Table 3:** Presenting symptoms, imaging and clinical considerations in relation to site and causes of large bowel perforation

Site	Presentation	CT findings	Causes	Cause-specific findings	Considerations
Colorectal	Abdominal pain, nausea, anorexia, vomiting, fever, sepsis	IP gas (cecum, transverse, sigmoid, upper 2/3 of rectum), EP gas (ascending, descending colon, lower 1/3 rectum), extraluminal faecal contents, oral/rectal contrast leakage, wall defect, faecal material protruding through wall/lying within abdominal cavity, bowel wall thickening (> 5 mm), fat stranding, abnormal wall enhancement, abscess, inflammatory mass adjacent to colon, free fluid	Tumour	Wall thickness > 1.39 cm, irregular wall configuration, lymphadenopathy, metastatic disease, free gas, minimal in tumour necrosis, free gas massive following obstruction	Tumour necrosis/following obstruction
			Iatrogenic	Disproportionate amount of extraluminal gas, stent extending through wall defect	History of instrumentation, opioids, radiation therapy, NSAIDs, chemotherapeutic regimens, corticosteroids
			Spontaneous	Caecal diameter > 14 cm, diffuse bowel dilatation without transition point	Severely ill, postoperative patients
			Diverticulae	Inflamed diverticulum, pneumoretroperitoneum	
			Trauma Foreign body	Foreign body, colovesical fistula, inflammatory mass	
			Stercoral	Faecal impaction with wall thickening, Faecaloma protruding through colonic wall/in abdominal cavity	Elderly, chronic costipation, scleroderma, bedridden patients
			Infectious		Salmonella, yersinia, tuberculosis, amoebiasis, Cl. difficile, E. coli, schistosomiasis, shigellosis, herpes, gonorrhoea, syphilis, LGV, CMV
			Ischemia	Poor/absent mural enhancement, pneumatosis intestinalis, vascular occlusion, portomesenteric gas	Low-flow states, vascular occlusion
			IBD	Skip lesions, intramural fat, fistula formation, marked colonic dilatation in UC	Free perforation rare

*EP* extraperitoneal perforation, *IP* intraperitoneal perforation, *NSAIDs* non-steroid anti-inflammatory drugs, *LGV* lymphogranuloma venereum, *CMV* cytomegalovirus, *IBD* inflammatory bowel disease, *UC* ulcerative colitis

## Conclusions

While the diagnosis of GIT perforation may be straightforward based on clinical and radiographic findings, the contribution of CT is twofold: subtle findings not visible on x-rays may be revealed, therefore supporting the diagnosis and directing appropriate management. Additionally, ancillary findings not necessarily related to the perforation may suggest underlying conditions that need further investigation following primary repair of ruptured bowel. Knowledge of the heterogeneous causes of GIT perforation is important for both the surgeon and the radiologist in order to ensure optimal management.

## Data Availability

Data sharing is not applicable to this article as no datasets were generated or analysed during the current study.

## Abbreviations

CD: Crohn’s disease
CT: Computed tomography
ERCP: Endoscopic retrograde cholangiopancreatography
GISTs: Gastrointestinal stromal tumours
GIT: Gastrointestinal tract
iv: Intravenous
MDCT: Multidetector computed tomography
NSAIDs: Non-steroid anti-inflammatory drugs
PUD: Peptic ulcer disease
